# Microwave-Assisted Organic Synthesis: An Eco-Friendly Method of Green Chemistry

**DOI:** 10.3390/ph18111692

**Published:** 2025-11-07

**Authors:** Josè Starvaggi, Roberta Ettari

**Affiliations:** Department of Chemical, Biological, Pharmaceutical and Environmental Sciences, University of Messina, Viale Ferdinando Stagno d’Alcontres 31, 98168 Messina, Italy; jose.starvaggi@studenti.unime.it

**Keywords:** microwaves, green chemistry, green solvents, green reagents, chemical synthesis

## Abstract

The name Green Chemistry was coined in 1996 to point out the development of chemical substances and sustainable processes that reduce the formation of toxic products for the environment and humans. The urgent need to bring down the negative effects of the chemical industry to safeguard human health has been the driving force behind green chemistry and the need to respect the United Nations Sustainable Development Goals. This approach allows to increase the effectiveness of synthetic methods, to develop safer, less toxic, and environmentally sustainable chemicals. In this context, microwave-assisted organic reactions revolutionized the chemical synthesis; as a matter of fact, microwave chemistry led to a low environmental impact of the used solvents, and, over the years this overture has become the method of choice in synthetic chemistry. This review highlights in detail the main features of microwaves.

## 1. Fundamentals Principles of Microwave-Assisted Organic Synthesis

Microwave-assisted organic synthesis (MAOS) is the part of organic chemistry which applies microwave radiation to chemical reactions, where MAOS was firstly developed in 1986. In the last 30 years, microwaves represented a fundamental turning point in synthetic chemistry with the aim to increase the reaction rate and yields and reduce by-products formation. MAOS was first reported in 1986 in two independent studies. Gedye and co-workers in Canada, and Giguere, Majetich, and colleagues in the United States, demonstrated that organic reactions performed in domestic microwave ovens could be dramatically accelerated, often with higher yields and cleaner profiles compared to conventional heating methods [1,2].

These pioneering reports marked the birth of MAOS, although early adoption was limited due to safety concerns, poor reproducibility, and the lack of equipment specifically designed for chemical applications.

During the late 1980s and early 1990s, further investigations highlighted both the promise and the limitations of the technique [3].

A major breakthrough came in the mid-1990s with the introduction of dedicated microwave reactors, which provided precise control over temperature, pressure, and power. This technological advancement enabled systematic studies of microwave effects and expanded the scope of transformations that could be reliably performed under microwave irradiation.

By the early 2000s, MAOS matured into a widely accepted methodology, with comprehensive reviews and mechanistic discussions consolidating its theoretical foundations and practical advantages [4].

Since then, the technique has been applied across diverse areas of chemistry, including heterocyclic synthesis, peptide chemistry, polymer science, materials chemistry and is now recognized as an important tool for green and sustainable synthesis due to its reduced reaction times, energy efficiency and lower solvent consumption [5].

This methodology is included in the field of green chemistry because there is an urgent need to pay attention to the environment, in fact, solvents that are normally used are less polluting such as water, which is an excellent solvent to carry out microwave assisted reactions [6]. Microwave (MW) irradiation is widely recognized as a green and sustainable method in organic synthesis because it addresses multiple principles of green chemistry. MW heating delivers energy directly and volumetrically to reactants, dramatically reducing reaction times and lowering energy consumption. Reactions often proceed under milder conditions with improved yields and selectivity, minimizing by-products and chemical waste. MW-assisted processes are compatible with solvent-free or low-toxicity solvent systems, reducing the use of hazardous solvents and aligning with the use of safer reaction media. The rapid and uniform heating allows for precise temperature control, decreasing risks of decomposition or thermal runaway and promoting inherently safer chemistry. Additionally, MW synthesis can facilitate catalytic or multi-component reactions, further enhance atom efficiency and reduce reagent consumption. Its high efficiency and selectivity reduce the need for extensive purification steps, cutting down on energy and resource use. By integrating energy savings, waste reduction, safer reaction conditions, atom efficiency, and minimal solvent use, MW-assisted organic synthesis exemplifies a holistic application of green chemistry principles, offering a practical, environmentally friendly, and sustainable alternative to conventional thermal methods [7,8].

While it is correct to state that MAOS falls within the scope of green chemistry due to its reduced environmental footprint, this statement can be strengthened by illustrating concrete cases in which MAOS provides measurable ecological benefits compared to conventional heating methods. For instance, Kappe et al. demonstrated that microwave irradiation significantly reduces reaction times from hours to minutes in heterocyclic synthesis, thereby lowering overall energy consumption and minimizing waste generation [9].

Similarly, Varma et al. reported solvent-free or aqueous-based microwave protocols for a variety of organic transformations, highlighting a clear reduction in the use of toxic organic solvents and improved atom economy [10,11].

Another illustrative case is found in peptide chemistry, where microwave-assisted solid-phase synthesis affords higher yields and shorter cycle times with reduced solvent consumption compared to conventional methods [12].

These examples underscore how MAOS not only accelerates reactions but also concretely contributes to greener processes through lower energy demand, reduced solvent usage and enhanced efficiency, aligning with the principles of sustainable chemistry.

The term green chemistry has received widespread attention and describes the development of chemical products and processes that reduce the use of toxic and carcinogenic substances for human health [13,14]. Green chemistry, as delineated by Anastas and Warner, encompasses twelve principles aimed at reducing or eliminating the use and generation of hazardous substances in the design, manufacture, and application of chemical products. Recent literature underscores the continuous evolution of this field, highlighting innovations such as the development of green solvents, renewable feedstocks, and energy-efficient processes [15].

These advancements are pivotal in steering the chemical industry towards more sustainable practices. MAOS has emerged as a significant technique within green chemistry, offering several advantages over conventional heating methods. The application of microwave irradiation facilitates rapid and uniform heating of reaction mixtures, leading to enhanced reaction rates, higher yields, and improved selectivity. These benefits are particularly pertinent in the context of green chemistry, where efficiency and sustainability are paramount. From a green chemistry perspective, MAOS offers several environmental benefits. By facilitating solvent-free reactions or enabling the use of safer solvents like water or ionic liquids, MAOS reduces the reliance on hazardous and toxic solvents. Moreover, MAOS often enables solvent-free reactions, thereby reducing the environmental impact associated with solvent use and disposal. The technique also allows for precise control over reaction conditions, which can be crucial in minimizing by-products formation and reducing energy consumption. Recent advancements in MAOS further expanded its applicability and sustainability. For instance, the development of microwave-transparent reaction vessels has enabled the design of continuous-flow microwave reactors, allowing for scalable and reproducible synthesis under controlled conditions. Moreover, the integration of microwave irradiation with other green methodologies, such as biocatalysis and photocatalysis, has led to the development of hybrid systems that offer enhanced selectivity and efficiency [16].

By aligning with the principles of green chemistry, MAOS not only enhances the efficiency and selectivity of chemical reactions but also contributes to the overarching goal of reducing the environmental footprint of chemical processes.

Consequently, microwaves (MW) are considered as an important approach toward green chemistry because MW-assisted chemical syntheses are more eco-friendly with a low environmental impact.

MW-assisted chemical reactions are based on the dielectric heating of molecules, but, unfortunately, not all reactions can be carried out under microwave irradiation, and its successful application depends on the nature of the reactants, solvents, and reaction conditions. MAOS is particularly effective for reactions that involve polar solvents or reagents with high dielectric constants, which efficiently absorb microwave energy and convert it into heat; this allows for rapid and uniform heating, often resulting in shorter reaction times, higher yields, and improved selectivity [17].

Examples of reactions suitable for MAOS include the following: (i) cyclization reactions, e.g., the synthesis of quinolines via the Friedländer reaction can be completed in 5–10 min with yields above 85% under microwave conditions; (ii) heterocyclic syntheses, e.g., coumarins, pyrazolopyrimidines, and imidazole are efficiently prepared under microwave irradiation.

Examples of reactions less suitable for MAOS include the following: (i) reactions in non-polar solvents, e.g., reactions in hexane or toluene often proceed inefficiently because these solvents poorly absorb microwave energy; (ii) highly exothermic reactions or those with sensitive functional groups, e.g., pericyclic reactions or reactions involving diazonium salts can be unsafe under microwave heating.

Microwave heating is a form of dielectric heating that utilizes electromagnetic waves within the frequency range of 0.3 GHz to 300 GHz [18].

The primary mechanism of microwave heating involves the interaction between oscillating electric fields and polar molecules. Polar molecules possess a permanent dipole moment, allowing them to align with alternating electric fields. As the frequency of the applied electric field matches the relaxation time of the dipoles, efficient energy absorption occurs, leading to rapid molecular rotation and subsequent heat generation.

The frequency range of microwaves is particularly effective for inducing dielectric heating in polar substances. Within this spectrum, frequencies between 0.3 GHz and 300 GHz are used, with common industrial applications operating at specific frequencies such as 2.45 GHz, which balances penetration depth and heating efficiency.

Incorporating these theoretical insights into the discussion would provide a more robust understanding of the principles governing microwave-induced dielectric heating, thereby enriching the content for readers seeking a deeper comprehension of the subject.

The essential requirement is that the molecules that react are polar, therefore, either the reagents are polar, or the solvent is polar, or better, all reagents and solvents. Microwaves have wavelength λ ranging from 1 mm to 1 m, corresponding to the frequencies between 0.3 GHz and 300 GHz, so they are located between the infrared and the TV waves. Microwave energy in chemical reactors is primarily generated by a magnetron tube, a vacuum device that converts high-voltage electrical energy into coherent electromagnetic radiation at a nominal frequency of 2.45 GHz, which is commonly used for laboratory and industrial applications due to regulatory allocations and optimal penetration depth in polar solvents. The magnetron emits microwaves into a resonant cavity, which serves as a reaction chamber and acts to confine and distribute the electromagnetic field around the sample. The cavity design is critical: its geometry, dimensions, and reflective surfaces determine the formation of standing waves, which affect the uniformity of microwave energy absorption and heating within the reaction vessel [19,20].

Modern microwave reactors incorporate an autotuning cavity system, which continuously monitors the reflected power from the reaction vessel and dynamically adjusts impedance-matching elements, such as movable plungers, variable capacitors, or dielectric tuners to maximize energy transfer from the magnetron to the sample. This prevents excessive reflected power that can damage the magnetron and ensures that the microwave field is efficiently coupled into the reaction medium.

In addition to autotuning, precise frequency control of the magnetron output is essential. Small deviations in frequency can shift the positions of nodes and antinodes within the cavity, leading to non-uniform energy distribution, localized hot spots, and variable reaction rates. Some advanced systems integrate frequency stabilization circuits or even variable-frequency magnetrons to optimize energy absorption for different solvent systems or reaction scales [21,22].

Together, these design elements, magnetron-based microwave generation, resonant cavity geometry, autotuning impedance matching, and frequency stabilization enable uniform volumetric heating, improved reaction kinetics, enhanced reproducibility, and safe scale-up of microwave-assisted chemical processes. Such careful engineering is particularly important for sensitive reactions, heterogeneous mixtures, or scale-up applications where precise control of energy input directly affects reaction selectivity and yield.

**Figure 1:**
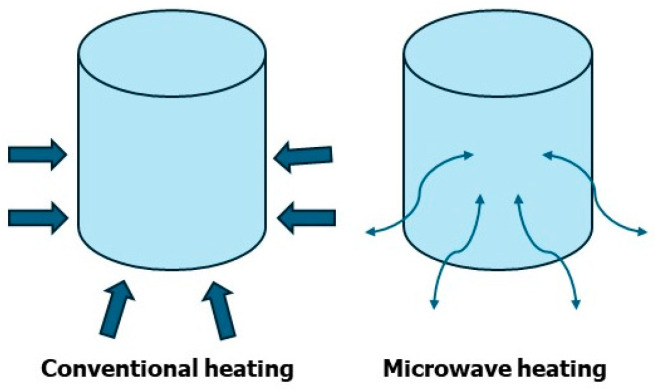
Microwave heating vs. conventional heating.

Normally, conventional synthesis, also known as batch synthesis, are carried out by heating the flask with a heating mantle; in this situation the heating starts from the outside of the reaction flask and reaches the center only in a sequential manner (Figure 1). This traditional heating is not homogenous, on the contrary, in the MAOS the heating is simultaneous in each part of the reaction mixture; this means that it occurs at the same time in all parts of the reaction mixture (Figure 1). MAOS offers distinct advantages over other alternative synthesis techniques, such as ultrasonication and mechanochemistry, primarily in terms of reaction efficiency and energy transfer. Unlike conventional heating, MAOS provides direct internal heating through the interaction of microwaves with polar molecules or ions, resulting in rapid and uniform temperature elevation that significantly accelerates reaction rates. In contrast, ultrasonication relies on acoustic cavitation, wherein the formation and collapse of microbubbles generate localized high temperatures and pressures, producing mechanical effects that enhance mass transfer and facilitate certain chemical transformations. While ultrasonication is particularly effective for heterogeneous reactions and nanoparticle synthesis, it generally requires longer reaction times and often produces less uniform heating compared to MAOS. Mechanochemistry, which employs mechanical forces such as grinding or milling to drive chemical reactions, offers solvent-free conditions and excellent environmental benefits, aligning closely with green chemistry principles. However, mechanochemical reactions are typically limited by scalability issues and require precise control of mechanical energy input. Overall, MAOS uniquely combines rapid, volumetric heating with compatibility for both homogeneous and heterogeneous systems, often resulting in higher yields, shorter reaction times, and broader substrate scope compared to ultrasonication and mechanochemistry. Nonetheless, the choice of method depends on the specific reaction type, substrate properties, and sustainability considerations, highlighting the complementary roles of these alternative synthetic strategies in modern organic chemistry.

Microwave chemistry is based on electromagnetic waves at a low frequency of 2.45 GHz, and it was initially hypothesized that, taking into account a simple reaction: A + B = C, that the irradiation could influence the breaking of the chemical bonds of the reactants by promoting the formation of the product. However, this hypothesis has been later excluded; in fact, the microwaves emission frequency of 2.45 GHz is not sufficient to break the chemical bonds existing at the reagent level. Therefore, the heat transmission mechanism is completely different. As previously mentioned, the fundamental requirement for the molecules to be used in microwaves is that they must be polar, i.e., equipped with an electric dipole moment, so that as soon as the emission of the electromagnetic radiation arrives from the magnetron tubes inside the microwave reactor, the molecules immediately polarize into dipoles. In this situation, the dipoles try to align themselves with the oscillating electric field; when this happens, they produce friction, and during this time there is the emission of thermal energy, i.e., heat. This event can explain what generates homogeneous heating, also called indirect heating [19,21].

It is also important to evaluate the potential role of non-thermal effects, including the influence of electromagnetic fields on reaction kinetics and molecular orientation. While some early reports suggested that microwaves might enhance reaction rates beyond what could be explained by bulk heating alone, the majority of contemporary evidence indicates that thermal effects, rapid, localized heating, and selective energy absorption by polar solvents or reagents are the dominant contributors to rate acceleration. Experimental studies comparing microwave and conventional heating under isothermal conditions have consistently shown that, when the temperature is controlled accurately, reaction outcomes are largely equivalent, supporting the notion that the observed acceleration in MAOS is primarily a result of efficient dielectric heating. Nevertheless, subtle non-thermal contributions cannot be entirely excluded, particularly in reactions involving highly polar transition states, oriented dipoles, or solid-phase reactants in heterogeneous systems, where localized electric fields could influence molecular alignment or energy barriers. Techniques such as in situ temperature mapping, fiber-optic thermometry, and computational modeling have provided quantitative support for the predominance of thermal mechanisms, while highlighting that any non-thermal effects, if present, are typically secondary and context-dependent. This evidence underscores the importance of carefully distinguishing between true microwave-specific phenomena and conventional thermal effects when interpreting MAOS outcomes.

This chemical method shows advantages including:

- Higher reaction speeds compared to those in conventional heating, which occurs for example by heating the mixture in batch with the heating mantle and a bubble condenser. In MW conditions the heating speed is 4–8 °C per second [23]. For instance, the synthesis of bioactive heterocycles such as pyrroles, pyrazoles, and imidazoles has been significantly accelerated using microwave irradiation, reducing reaction times from several hours to just minutes while maintaining high yields [24].
- High reaction temperatures; in fact, the reactors work up to 300 °C. However, this is a limit temperature because if we set the reaction temperature at 300 °C we must consider many factors, like whether the reagents could degrade at those temperatures. Thus, generally, reaction temperatures are set until 150–160 °C, due to the instability of the reagents. Since microwave reactors work at high pressures (up to 30 bar), the increase in pressure inside the reactor allows to use much higher temperatures for each solvent, with respect to its normal boiling point (at 760 mmHg). However, the law that regulates the increase in temperature as a function of the increase of pressure is the Clausius–Clapeyron Equation (1) [23].
  (1) $$\ln\left(\frac{P1}{P2}\right)=\;\Delta H_{\mathrm{vap}/}R\;(\frac{1}{T2}-\frac{1}{T1})$$
- High yields. The yields are always improved than in batch synthesis, due precisely to homogeneous heating; thereafter, reactions proceed with high reaction speeds, and obviously, if the yield is improved, the by-products are lower [23]. In peptide chemistry, MAOS methods facilitate rapid peptide bond formation under milder conditions, minimizing side reactions and the use of large volumes of solvents. For instance, a study demonstrated that MAOS peptide synthesis could achieve 68% crude purity of a complex peptide in under four hours, compared to traditional methods requiring 20 h [25].
- Minimization of the quantities of solvents. This problem is due to an economic point of view because the vials used in this apparatus are small, the sizes are 10, 35, and at most 100 mL, and so, the amount of solvent used is very small compared to traditional synthesis [23].
- Green solvents are used, solvents that have very low polluting potential, such as water, ethanol, methanol or ethylene glycol, which is why MW-assisted reactions move in the field of green chemistry. Common green solvents in MAOS include water, ethanol, glycerol, and polyethylene glycol (PEG). Water, being non-toxic and abundantly available, minimizes hazardous waste and reduces the reliance on volatile organic compounds (VOCs). Ethanol, derived from renewable biomass, offers low toxicity and biodegradability, making it an environmentally friendly alternative to conventional organic solvents. Glycerol and PEG are non-volatile, biodegradable, and reusable, contributing to lower environmental impact and enhanced process sustainability. Including such details would underscore the environmental benefits of these solvents, such as reduced toxicity, decreased flammability, and improved energy efficiency, thereby reinforcing the green chemistry principles applied in MAOS [23].

Other advantages of microwave-assisted reactions include the following:

(1) Safer working conditions, because since the system is automated, if an over pressure occurs, the reaction is stopped and the system subjected to venting [23].
(2) Reproducibility of the reactions carried out under microwave irradiation, for example, repeating a synthesis in which it seems that there is a variation in yield from one researcher to another. This problem is due to the operator variability, naturally, in the microwave reactor the processes are more reproducible, and the yields will certainly be much more comparable, because the automated procedures reduce human error and improve reproducibility by providing rapid, uniform heating, and precise control over reaction conditions. The incorporation of automated systems into MAOS further enhances these advantages by allowing for the precise programming of temperature profiles, irradiation power, and reaction times, which are critical parameters influencing reaction outcomes. Modern automated MAOS platforms, such as automated microwave reactors equipped with robotic sample handling and in-line analytical monitoring (e.g., IR, NMR, or mass spectrometry), enable high-throughput screening and parallel synthesis with minimal operator intervention. This not only standardizes reactions across multiple experiments but also facilitates the rapid optimization of reaction conditions, reducing trial and error approaches typical of manual synthesis. Moreover, automated data acquisition and process logging improve reproducibility and allow for better reaction tracking and troubleshooting. The synergy between microwave irradiation and automation thus provides a highly efficient, scalable, and reliable approach for both routine and exploratory organic synthesis, making MAOS a valuable tool in medicinal chemistry, heterocyclic synthesis, and materials science.
(3) This technique allows to work on both small or large scales; accordingly, there are both classic reactors specifically for synthesis in academies and then there are reactors for scale-up, which are those used at an industrial level [23]. MAOS has demonstrated significant advantages in enhancing reaction rates, improving yields, and reducing energy consumption at the laboratory scale. However, translating these benefits to industrial-scale synthesis requires careful consideration of several factors. The scalability of MAOS is influenced by the uniformity of microwave energy distribution, the thermal conductivity of the reaction mixture, and the ability to maintain consistent reaction conditions over extended periods. To address these challenges, continuous-flow microwave reactors have been developed, offering improved heat and mass transfer characteristics compared to traditional batch systems. These reactors facilitate the efficient processing of larger volumes, enabling the application of MAOS in industrial settings. For instance, a study demonstrated the successful scale-up of a continuous-flow microwave reactor operating at high temperatures and pressures, achieving throughput rates suitable for industrial applications. Additionally, the integration of Process Analytical Technology (PAT) with microwave systems allows for real-time monitoring and control of reaction parameters, ensuring consistent product quality and facilitating regulatory compliance [26].

Despite these advancements, challenges remain in the uniformity of microwave energy distribution and the design of reactors capable of handling the specific requirements of industrial-scale operations. Continued research and development are essential to optimize reactor designs, enhance scalability, and fully realize the potential of MAOS in industrial synthesis.

Hence, the microwave vials, which are called vessels, have a size of 10 mL (which can be filled up to half, up to 5 mL to leave space to increase pressure within the system) or 35 mL. The vial septum is hermetic with the aim of preventing opening during the reaction. The internal part of the vial cap is covered with a silicone septum internally coated with Polytetrafluoroethylene (PTFE), a synthetic fluoropolymer of tetrafluoroethylene [23]. Therefore, it is essential to highlight the specificity of microwave heating in organic synthesis, as its unique interaction with reactants supports the acceleration of chemical transformations. Microwave energy couples directly with polar molecules and ionic species through dipolar polarization and ionic conduction mechanisms, leading to rapid and homogeneous volumetric heating within the reaction medium. Unlike conventional thermal methods, which rely on slow conductive or convective heat transfer, microwaves induce molecular rotation and ion oscillation, thereby generating heat internally and selectively in regions with higher dielectric loss. This localized and efficient energy transfer not only shortens reaction times but can also promote reaction pathways that are less accessible under traditional heating conditions. Moreover, the enhanced kinetics often observed in microwave-assisted processes arise from the combination of precise temperature control, minimization of thermal gradients, and, in some cases, specific non-thermal microwave effects that remain a subject of scientific debate. Collectively, these mechanisms contribute to improved yields, cleaner reaction profiles, and the development of more sustainable synthetic protocols.

Despite the numerous advantages of MAOS, the technique is not without limitations and challenges that warrant critical consideration. One primary constraint is the scalability of MAOS, as uniform heating becomes increasingly difficult in larger reaction volumes, potentially leading to inconsistent yields or selectivity. Additionally, the high initial cost of specialized microwave reactors and the need for microwave-transparent or pressure-resistant vessels may limit accessibility for some laboratories. Certain reactions, particularly those involving non-polar or poorly microwave-absorbing substrates, may exhibit reduced efficiency, necessitating the use of additives or alternative solvents. Safety concerns, including the management of high pressures and rapid heating, also require careful protocol optimization. Future research should focus on the development of continuous-flow microwave systems, improved reactor design for large-scale synthesis and hybrid techniques that combine microwaves with other green methodologies, such as photochemistry or biocatalysis. Moreover, mechanistic studies aimed at elucidating the effects of microwave irradiation on reaction pathways could provide fundamental insights to expand the applicability of MAOS across diverse chemical transformations. Addressing these challenges will be essential to fully realize the potential of MAOS as a sustainable and versatile tool in modern organic synthesis.

**Figure 2:**
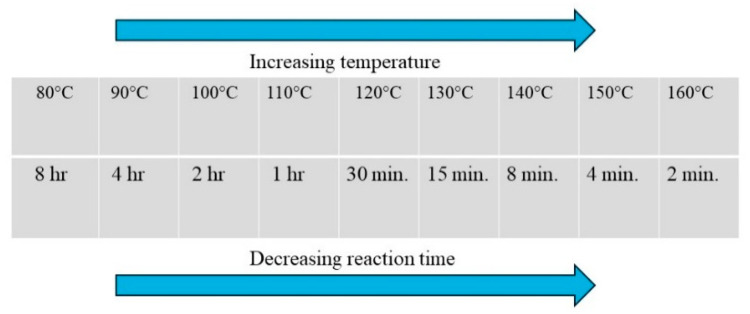
Thumb rule.

The theoretical basis to convert a batch reaction to a microwave-assisted synthesis is explained by Thumb rule (Figure 2) [27]. In MAOS, a widely acknowledged empirical guideline suggests that microwave irradiation can reduce reaction times by approximately an order of magnitude compared to conventional heating methods, while maintaining comparable yields and selectivity. This heuristic is grounded in the principles of dielectric heating, where microwave energy couples directly with polar molecules or ions in the reaction medium, resulting in rapid and volumetric heating. Unlike conventional conduction or convection heating, which relies on heat transfer from the vessel walls, microwave energy can produce localized superheating and non-equilibrium temperature distributions at the molecular level, thereby accelerating reaction kinetics.

However, the applicability of this “thumb rule” is constrained by several factors. The efficiency of microwave heating is influenced by the solvent’s dielectric properties, the geometry of the reaction vessel, and the scale of the reaction. For instance, solvents with low dielectric constants may not absorb microwave energy effectively, leading to inefficient heating. Additionally, the microwaves penetration depth is limited, which can result in uneven heating, especially in larger reaction volumes.

Furthermore, not all reactions uniformly respond to microwave irradiation. Some reactions may exhibit enhanced rates, while others may show no significant difference or even decreased yields under microwave conditions. This variability underscores the importance of empirical optimization and mechanistic studies when adapting traditional reactions to microwave-assisted methods [28].

Therefore, while the “thumb rule” serves as a useful starting point, it is essential to consider the specific characteristics of each reaction system. Systematic experimentation and a thorough understanding of the underlying principles of microwave heating are crucial for successfully translating batch reactions to microwave-assisted synthesis.

This guideline helps the researcher to understand how to set up a microwave reaction starting from a traditional heating reaction; thus, considering a batch reaction conducted at 80 °C for 8 h if we apply an increase of 10 °C to the reaction time (i.e., 90 °C), the reaction will work in 4 h. If the reaction will be conducted at 100 °C it will take 2 h. Clearly, this principle is useful to understand how to translate a batch synthesis reaction into a microwave-assisted reaction. Generally, microwave-assisted reactions are never carried out below 100 °C to take advantage of high-temperature effect.

Several case studies demonstrated its applicability and effectiveness across diverse reaction classes. For instance, the synthesis of nitrogen- and oxygen-containing heterocyclic scaffolds under microwave conditions has consistently shown significant reductions in reaction times, often by an order of magnitude, while maintaining comparable yields and selectivities relative to conventional heating [29].

This example highlights not only the practical utility of the thumb rule but also its versatility across different solvents, catalysts, and reaction scales. Nevertheless, the outcomes of microwave-assisted reactions remain sensitive to factors such as solvent polarity, vessel geometry, and reaction concentration, underscoring the importance of empirical optimization in each case. By providing concrete examples and case studies, researchers can better understand the scope and limitations of the thumb rule, thereby enhancing its reliability in the design of efficient MAOS protocols. Furthermore, thumb rule can also provide guidance in more complex reaction systems, such as multi-step syntheses or reactions involving multiple reagents. In these contexts, the rule is applied iteratively, with each individual step evaluated according to established principles such as kinetic favorability, thermodynamic stability, or functional group compatibility. While the rule does not replace detailed mechanistic analysis or computational modeling, it serves as a practical tool for prioritizing reaction pathways, selecting suitable reagents, and anticipating potential side reactions. Furthermore, in multi-step syntheses, the cumulative effect of successive transformations can be approximated using the thumb rule, enabling chemists to identify sequences that are likely to proceed efficiently while minimizing undesired byproducts. Nonetheless, the predictive power of the rule diminishes as system complexity increases, necessitating complementary approaches such as experimental screening or theoretical calculations.

In the microwave, a key role is played by the type of solvent used to conduct the reaction and clearly, not all solvents are suitable to be used in a microwave system. Until some years ago the most important parameter to be considered for solvents was the dielectric constant ε′, that indicates the ability of a solvent to polarize in the presence of electromagnetic radiation. Referring exclusively to the dielectric constant, the best solvent was water with a dielectric constant of 78.3 (Table 1), while ethanol shows a dielectric constant of 24.3 and methanol of 32.6; thus, from a first examination water seems to be the best solvent to use in MAOS.

Currently, the primary parameter to select a solvent to be used in MAOS is not the dielectric constant, but the tangent δ, that is the ratio of ε″/ε′, where ε″ is the dielectric loss and essentially indicates the ability of a solvent to dissipate the electromagnetic energy under heating. The tangent δ, also called dissipation factor, indicates the capacity of a solvent to convert the electromagnetic energy in the form of heat because it incorporates both the dielectric loss and the dielectric constant [30,31]. The table below (Table 2) classifies values of tan δ for solvents as high (tan δ > 0.5), medium (0.1 > tan δ < 0.5), and low microwave absorbing solvents (tan δ < 0.1), and within solvents with tan δ >0.5 and the absolute best solvent is ethylene glycol (tan δ = 1.350), followed by ethanol (tan δ = 0.941) and methanol δ (tan δ = 0.659), while water show a medium tan δ = 0.123.

Ethylene glycol (EG) is a high-boiling, polar diol that has been extensively utilized as a solvent in MAOS due to its exceptional dielectric properties. With a dielectric constant of approximately 37 at 25 °C and a high dielectric loss tangent (tan δ ≈ 1.35 at 2.45 GHz), EG efficiently absorbs microwave energy, converting it into rapid and uniform thermal heating. This property enables accelerated reaction kinetics, often reducing reaction times from hours to minutes compared to conventional heating methods. In addition to its dielectric characteristics, EG exhibits a strong hydrogen-bonding capability, which can stabilize polar transition states and reactive intermediates, thus influencing reaction selectivity and yield. However, its chemical reactivity must be carefully considered; the hydroxyl groups of EG can act as nucleophiles or reducing agents under certain conditions, potentially leading to side reactions such as ether formation, esterification, or oxidation products. To minimize these risks, reaction parameters including temperature, microwave power, reaction time and stoichiometry are carefully optimized, and inert atmospheres or stabilizing additives may be employed. Analytical monitoring using NMR, HPLC, or mass spectrometry is essential to detect trace byproducts and confirm that EG functions primarily as an inert solvent. The high boiling point of EG also allows reactions to be conducted at high temperatures under microwave irradiation without significant solvent loss, making it particularly suitable for high-temperature transformations, cyclizations, or condensations in organic synthesis. Overall, EG combines excellent microwave absorption with thermal stability, offering both kinetic and mechanistic advantages, provided that its potential reactivity is carefully controlled. Clearly, reactions could be carried out in water, which is why MAOS is considered a part of green chemistry, but ethanol and methanol are also green solvents with a low environmental pollution. Instead, the solvents that have a low delta tangent (less than 0.1) are all non-polar solvents, such as chloroform, dichloromethane, toluene, and hexane, which are those that did not polarize. For example, olefin metathesis reactions can be carried in a microwave reactor; however, these reactions are 2 + 2 cycloadditions and could occur if the metallo-cyclobutane is well solvated, and this happens with dichloromethane (DCM). However, this will imply a longer run time, due to poor polarization of DCM, and thus the heating of the mixture can be obtained only if reagents are polar. As previously mentioned, the way with which a material will be heated by microwaves depends on its polarity, dielectric constant, and by the nature of the microwave tools used. In microwave irradiation, the main mechanism for dielectric heating is dipolar loss, also known as the re-orientation loss mechanism. When material containing permanent dipoles is subject to a variable electromagnetic field, the dipoles are unable to follow the rapid reversals in the field. As a result of this phase, power is dissipated in the material under form of heat [32,33]. In MAOS, the dielectric constant of a solvent is a key parameter influencing microwave absorption and heating efficiency; however, it is not the sole factor determining reaction outcomes. Solvent polarity and hydrogen bonding are critical factors influencing reaction mechanisms and outcomes. While the dielectric constant of a solvent determines its microwave absorption efficiency, solvent polarity affects the stabilization of transition states and intermediates, thereby influencing reaction rates and selectivity. For instance, in nucleophilic substitution reactions and polar aprotic solvents like dimethyl sulfoxide (DMSO) enhance the nucleophilicity of anions by solvating cations, leading to increased reaction rates compared to polar protic solvents such as ethanol, which can form hydrogen bonds with nucleophiles, reducing their reactivity.

Hydrogen bonding further modulates reaction pathways by stabilizing specific conformations or transition states. For example, in the synthesis of heterocyclic compounds, solvents capable of hydrogen bonding can stabilize polar transition states, leading to higher regio- and stereo-selectivity. Conversely, in reactions where hydrogen bonding could lead to side reactions or decomposition, selecting a solvent with minimal hydrogen bonding capability is advantageous.

Additionally, the solvent’s ability to engage in hydrogen bonding can influence the solvation of reactants and intermediates, affecting the overall reaction mechanism. For example, in the synthesis of organophosphorus compounds, the choice of solvent can impact the formation and stability of reactive intermediates, thereby affecting the reaction’s efficiency and product distribution [33].

These examples underscore the importance of considering solvent polarity and hydrogen bonding in the design and optimization of MAOS processes. A comprehensive understanding of these factors enables chemists to tailor reaction conditions for desired outcomes, enhancing the efficiency and selectivity of microwave-assisted reactions.

Microwave reactions can be conducted in the following:

(a) **In solution**, where the reagents go into solution in the solvent.
(b) **Supported reagents** could be used; indeed, these are particularly frequent both in these types of reactors and especially in flow chemistry ones. In this case, reagents are anchored to support, which can usually be of polymeric nature, or composed by silica or an alumina matrix. The advantage is that if there is a supported reagent A and reagent B in solution the product will remain linked to the support and will be recovered by filtration. So, it is clear that the use of supported reagents will make the purification processes easier, both in microwave and flow chemistry reactors [33]. The use of supported reagents represents a valuable strategy to enhance reaction efficiency, facilitate product isolation, and minimize solvent usage. Ensuring uniform distribution of the reagent on the support material is critical in achieving reproducible and selective outcomes and this is typically accomplished through controlled impregnation, co-precipitation, or deposition techniques that maximize surface area exposure. Characterization methods such as scanning electron microscopy (SEM), energy-dispersive X-ray spectroscopy (EDX) and infrared spectroscopy are often employed to verify homogeneity and chemical integrity of the supported systems. Equally important is the selection of an inert support, such as silica, alumina, or polymeric matrices, to prevent undesired interactions with the reagents or substrates; in cases where the support possesses catalytic properties, it must be carefully matched to the target transformation to avoid competing pathways. Despite these advantages, the use of supported reagents does present limitations, not all reaction types are compatible, particularly those requiring homogeneous conditions or involving bulky substrates with limited access to the reactive sites. Additionally, reusability of the supported system may decrease over successive cycles due to leaching or degradation of the active species. These considerations highlight both the opportunities and the challenges of integrating supported reagents into MAOS, underscoring the need for tailored design of both reagents and supports for each specific application.
(c) If one reagent is used in excess, **specific scavengers** can be used to selectively capture the reagents in excess. Considering a 1 equivalent of reagent A and 1.5 of reagent B, the scavenger is used for the half equivalent in excess of reagent B; therefore, in the subsequent filtration the scavenger linked to the excess of the reagent B will remain on the filter, while the reaction product will be recovered solution [33].
(d) **Atmosphere:** Reactions can be carried out under gaseous atmosphere, using hydrogen rather than inert gas and others. The vials, that will be appropriately inserted into the reactor, are hermetically closed with a PTFE septum, if a reaction under gaseous atmosphere has to be carried out a “gas addiction kit”, will allow to first perform the vacuum into the vial and then to flow the gas of interest directly into the reaction mixture in absolute safe conditions [33].
(e) **Temperature:** the reactors start from 60 °C and reach up to 300 °C, even if the real working range of activity is between 100 °C and 150° C to avoid reagent decomposition.
(f) **Pressure**: In terms of pressure, the maximum that can be reached is 30 bar; obviously, the pressure control systems are automated, so that the reactors that are based on artificial intelligence (AI) normally calculate applying Clausius Clapeyron Equation (1) the real pressure required to avoid solvent evaporation at the selected temperature. If, during the reaction time, an overpressure occurs, the reactor stops immediately and vents, so systems are very safe compared to batch synthesis. As an example, if we consider acetone, which has a boiling point of 56 °C at atmospheric pressure, i.e., 1.01 bar that corresponds to 1 atm or 760 mmHg, setting the reaction in the MW reactor at 30 bar, the temperature can be selected until 183.5 °C without solvent evaporation. This law regulates the increase in temperature with the pressure of the solvents inside the reactor and describes that the logarithm of P1/P2 (P1 is 1.01 bar, P2 is 30 bar) is equal to the ratio between the enthalpy of vaporization and the gas constant (R = 8.314 J/(K mol)) multiplied for the difference of the ratios 1/T2−1/T1, where T1 is 56 °C, and T2 is the unknown parameter to find. Thus, solving this equation T2 results in 183.5 °C. Therefore, this calculation allows us to know what is the maximum ceiling beyond which the solvent evaporates even in the microwave reactor [31]. The advancement of MAOS has been closely linked to the integration of sophisticated monitoring and control systems that ensure reproducibility, safety, and scalability. Conventional thermocouple-based monitoring is often inadequate under microwave conditions due to electromagnetic interference and slow response times; consequently, fiber-optic sensors, infrared (IR) pyrometry, and sapphire-crystal probes have become the standard for real-time, non-invasive temperature measurements with high accuracy. Pressure monitoring is achieved through piezoelectric or strain-gauge transducers, enabling precise control of sealed-vessel systems operating at elevated temperatures, thus extending the accessible reaction window. These sensor inputs are processed by advanced control algorithms, typically employing proportional-integral-derivative (PID) regulation to continuously modulate microwave power output and maintain isothermal or ramped profiles. Beyond PID, model predictive control and machine learning (ML) based approaches are increasingly being explored to predict solvent-specific absorption, reaction kinetics and thermal inertia, thereby allowing pre-emptive adjustments rather than reactive corrections. In parallel, the incorporation of Process Analytical Technology (PAT) has transformed MAOS into a data-rich process: in situ Raman, NIR, and UV–Vis spectroscopic probes permit continuous monitoring of chemical transformations, providing mechanistic insights and enabling real-time feedback loops between reaction progress and power modulation. Such integrated sensor-algorithm frameworks not only minimize risks of thermal runaway and by-product formation but also facilitate scale-up from laboratory to industrial applications, aligning MAOS with modern principles of process intensification and quality-by-design.
(g) **Concentration of the reaction**. Another condition that has to be considered is the concentration of the reaction mixture, that is not specifically relevant if the reaction is unimolecular, while if the reactions are bi- or tri-molecular, the concentration of the reagents is very important for the yield and the speediness of the reaction [33].
(h) **Volume of the vials.** Another important factor in MAOS is the volume because we should never fill the vials more than half of their capacity, otherwise, there will be no space necessary for the pressure increase [33].

The microwave reactors are made up of magnetron tubes that are positioned in the autotuning cavity which irradiate the sample at 2.45 GHz. These are frequencies that did not allow to break the bonds of the reagents; therefore, they are simply promoters of the polarization of the molecules to ensure that these, in the attempt to align themselves with the oscillating electric field, produce frictions and disperse energy under form of heat. Obviously, the internal parts of these reactors must be made of materials that do not absorb themselves in the microwave’s energy, otherwise this emission will arrive in reduced manner on the sample. The most used materials are quartz, aluminium oxide, and then pyrex glass [33]. The precise overview of the temperature variation during reaction is due to specific sensors; the temperature is measured by optical fibers or infrared sensors. However, these sensors were present in the first generations of reactors. On the contrary, more recent reactors show *i*-wave sensors that use the new “light emitting technology” (LET), which is able to detect the temperature of the sample and not the temperature of the reaction container. MW reactors normally conduct reactions at pressures up to 30 bar in **a closed-vessel** or atmospheric pressure **in an open vessel** [34,35]. **Closed-vessel reactions** mean that the vial in which the reaction takes place is closed, and the system is under pressure. On the other hand, it is possible to conduct microwave reactions under atmospheric pressure; in this case reactions are in an **open vessel**, with the vial connected to a refrigerant. In this case, instead of using the heating mantle the mixture is heated by microwave irradiation. The microwave reactors are also equipped with a **cooling system**, which allows, once the run time of the chemical reaction is finished, to cool the system before releasing the vial, generally not before reaching 40 °C. Cooling occurs automatically for about 20 min; however, if the reactor is connected to a compressed air cylinder, the air is flowed into the autotuning cavity and cooling occurs quickly. Reactions can be performed by introducing into the vial a stirring bar to carry out the reactions under stirring, with the possibility to set the stirring speed as low, medium, or high. The first-generation reactors essentially did not have a touch screen on the reactor itself, but usually are connected with a scart socket to a computer on which there is installed the related software, thus allowing to see the variation of pressure, temperature, and power during the reaction time [34]. Differently, unlike the first-generation reactors, which used infrared or optic fibers as sensors, the latest generation reactors use the *i*-wave sensors based on the LET, which allows to measure the temperature directly inside the sample. This last generation of reactors also possess an integrated video camera, thus establishing visual contact between the internal cavity of the instrument and the operator. This modern technology allows us to shoot videos during the reaction time and take photos, which can then be found at the end of the run time in a specific section of the reactor. Different types of methods can be set with the latest generation reactors, the most used when we work in closed vessel is the dynamic method, which allows us to set the three main parameters of the reactor, i.e., pressure, temperature, and power. The pressure in these types of apparatus can be set to a maximum of 30 bar, but these artificial intelligences regulate the pressure according to the type of solvent in which the reaction was carried and its δ tangent, or by considering Clausius–Clapeyron equation. Regarding the power, if the solvent is polar (such as ethanol or methanol) it can be set to 70 W–100 W, while if the solvent is non-polar (e.g., dichloromethane) the power must be set in the range 150 W–200 W [34]. The reaction containers can be of 10 mL, 35 mL, or 125 mL for closed-vessel reactions, while 125 mL flasks are used for open-vessel reactions. The novelty of these latest generation reactors is that, without the need to indicate the capacity of the vials, the reactor recognizes the capacity of the reaction container from the type of adapter located in the microwave cavity. Differently, regarding the open-vessel reactions, in this case the method used for the run time is at fixed power; in this case, the microwave irradiation will allow the polarization of the molecules and to convert the kinetic energy under heating; however, the boiling points of the solvents remain those normal at atmospheric pressure [34].

**Figure 3:**
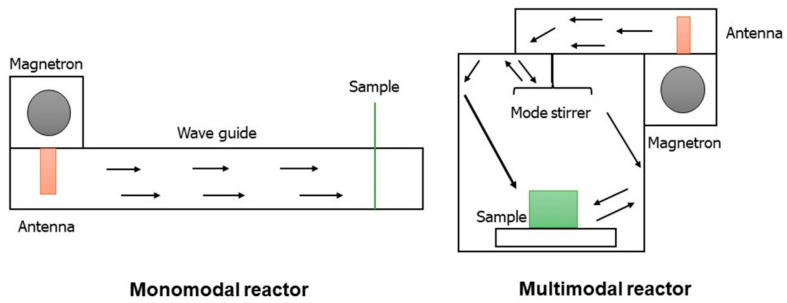
Single-mode and multi-mode reactors.

Microwave reactors are divided into two categories: **single-mode** and **multi-mode reactors** [35,36]. Single-mode reactors are the most common, and more than 99% of reactors are all single-mode; these reactors are made up of a single magnetron tube emitting an electromagnetic radiation that arrive directly on the sample via this “wave guide”, clearly depicted in Figure 3.

**Figure 4:**
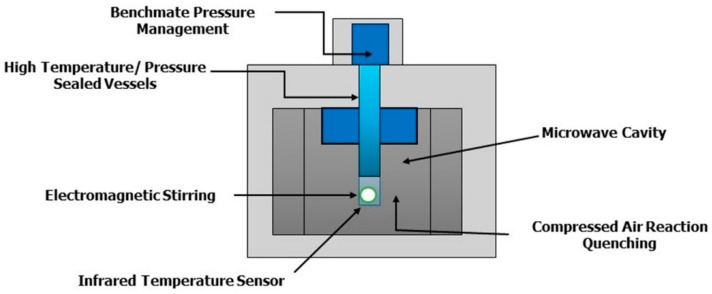
Microwave cavity.

The multi-mode apparatus [35,36] is widely used for parallel synthesis in which scale-up occurs. These reactors contain more than one magnetron tube because in the parallel synthesis we can put to react up to 32 vessels at the same time, and clearly, a single magnetron tube is not enough to irradiate all vials. From a geometric point of view, these radiations are partially reflected by the walls of the reactor. Inside this reactor there is a “mode stirrer”, which allows the radiation to reach the sample. That is why, in most cases the single-mode reactors are preferred. The power of the reactor reaches up to 1800 W, as we have multiple magnetron tubes to irradiate all these vessels. The stirring speed is eight rotations per minute. The cavity, in the single-mode reactors, is compact, while in the multi-mode reactor the cavities are roomy to accommodate up to 32 vials, in fact, there is a carousel with 32 vials and therefore the auto-tuning cavity is very large. In a closed-vessel, but also in an open vessel, in the single-mode reactors the maximum capacity of the reactor container is 125 mL, and in multi-modal reactors even up to 1 L. That is why it is used for scale-up synthesis. The single-mode reactor has lower emission power because it uses only one magnetron tube, while the multi-mode ones show a higher emission power [35,36]. However, the field density is greater in single-mode apparatus because the radiation arrives directly on the sample while in multimode reactor, as it is partially reflected by the walls of the reactor, the field density will be, all in all, lower. This is the reason why, except for parallel or scale-up synthesis, that the reactors are preferentially all in single-mode. Figure 4 shows the microwave cavity.

A balanced evaluation of single-mode and multi-mode microwave reactors is essential, as each design offers distinct advantages and disadvantages that directly impact reaction performance and product yields. Single-mode reactors deliver focused and uniform microwave irradiation, ensuring precise temperature control and reproducibility, which is particularly advantageous for mechanistic studies, optimization of sensitive reactions, and cases where selectivity is crucial. Their main limitations, however, lie in the restricted reaction volume and the challenges associated with scaling up, which limit their applicability in preparative or industrial contexts. On the other hand, multi-mode reactors enable the simultaneous processing of larger volumes and multiple samples, making them more suitable for high-throughput synthesis and scale-up experiments. Yet, the broader distribution of microwave energy in multi-mode systems often results in less homogeneous heating, the formation of hot spots and reduced control over reaction parameters, which may compromise reproducibility and selectivity [33]. The choice of reactor design therefore depends on the specific goals of the experiment; when high precision, control, and reproducibility are required, single-mode systems are generally preferred, whereas multi-mode systems are advantageous when throughput and scalability are prioritized. Ultimately, these differences have important implications for reaction outcomes, since single-mode reactors tend to provide higher consistency and selectivity, while multi-mode reactors offer efficiency in processing but may require additional optimization to achieve comparable yields and reproducibility. After the insertion in the microwave cavity of vials closed with the polytetrafluoroethylene (PTFE) septum, the pressurizer called benchmate pressure management closes automatically. Clearly, Figure 4 shows the microwave cavity where there is a magnetron tube that irradiates the reaction mixture and on the bottom of the apparatus, an infrared sensor is localized. Eventually, the connection with a compressed air cylinder on the back of the reactor, allows a faster cooling of the reaction mixture in 10 min instead of 20 min. The modern reactors have the possibility to add various modules, such as the autosampler with the aim to carry out multiple reactions in sequence, e.g., if we perform 12 metathesis reactions, each with a run time of 1 h, it is possible to put all the 12 reactions in the autosampler, where each reaction will be put to react one at a time [33]. At the same time, it is possible to insert several modules to carry out reactions under inert gas or simply hydrogenations. In this case it is necessary a “gas addition kit” which shows a pressure regulator for the gas pressure. Through one of the valves, it is possible to connect the gas addition kit with the hydrogen cylinder, by conducting the hydrogenation in absolute safe conditions. Alternatively, if we want to perform a reaction under inert gas, the gas addition kit has to be connected with a nitrogen or with a helium cylinder; of course, before flowing the gas into the vial, the vacuum is applied into the vial. In Table 3 a few examples are depicted, like the formation of an imine which is then hydrogenated or the reduction of nitro groups to amino groups. The integration of additional modules such as autosamplers, gas addition kits, and other auxiliary components into MAOS systems represents an important strategy for expanding functionality and enhancing experimental flexibility. Ensuring compatibility with existing reactor designs and control systems typically requires standardized hardware interfaces, robust communication protocols, and software integration that allows seamless synchronization of operational parameters, such as temperature, pressure, and microwave power. However, this process is not without challenges. Differences in reactor geometries, vessel configurations, and control architectures may restrict the straightforward incorporation of external modules, while maintaining reliable safety features, such as pressure release mechanisms and temperature cutoffs, can further complicate integration. Additionally, the addition of modules may increase system complexity, raising concerns related to calibration, maintenance, and potential sources of error. Limitations may also arise in terms of cost, as advanced modular systems can substantially increase the investment required, which may not be feasible in all laboratory contexts. Thus, while modular integration significantly broadens the scope of MAOS, it requires careful attention to system compatibility, reliability, and safety to ensure that the benefits outweigh the potential drawbacks.

It is also possible to carry out a reaction at sub-ambient temperature [37]. To perform a reaction at −78 °C the reactor is normally equipped with a cool-mate kit, which consists of a reservoir where liquid nitrogen or a perfluorinated liquid can be loaded, in addition there is an “in” and an “out” valve that allows the entry of the cryogenic liquid in the system, exactly in the external portion of the reaction container, cool the reaction and then return to the reservoir. Since this dissipation of energy in the form of heat is reduced by contact with the cryogenic liquid, therefore, microwave heating is used to increase the kinetic energy of the molecules, thus letting the reagents react quickly with an increase of the reaction yield. In addition, modern reactors are equipped to perform microwave-assisted solid phase synthesis, in this case the reactors no longer work at room temperature at 25 °C, but at very high temperatures.

These modern reactors perform a coupling cycle in 2.5 min, with respect to batch synthesis in which coupling can be performed in 12 h, because of the low kinetic of this type of reaction. These efficient reactors present 27 working stations to load the amino acids, the 20 natural aminoacids (L-configuration) and in addition it is possible to load unnatural aminoacids (D-configuration), which give greater enzymatic stability to proteolysis to the synthesized peptide. There are additionally two stations where it is possible to load the DIPEA and then coupling reagents, like HBTU or similar.

The most recent reactors show a 24-station autosampler, and in addition a five-channel pump system which even allows to inject four or five different external reagents within the reaction run time. This system is connected to software, because based on the positions of the amino acids, we have to set the exact sequence of the polypeptide chain that have to be synthesized.

## 2. Applications of Microwave Reactions

Microwave heating, widely known for cooking, has been successfully employed in various industrial and therapeutic applications, including nanomaterial synthesis [38,39,40,41], nanotechnology [42], and organic chemistry [43]. Notably, MAOS is primarily focused on pharmaceutical research and the development of new potential drugs, also known as drug discovery. The microwave-assisted synthesis of heterocyclic compounds offers several advantages in the pharmaceutical field compared to traditional synthetic protocols [44,45,46]. The construction of *N*-heterocycles [47] using microwave technology represents a sustainable approach to design novel molecules [48]. In MAOS of *N*-heterocycles, scalability and safety are crucial challenges. Industrial implementation requires optimized reactor design, with continuous-flow microwave systems offering improved heat transfer, reproducibility and safer operation compared to batch processes. In addition, strict safety protocols and the use of greener solvents help to minimize risks for operators and reduce environmental impact, making large-scale applications both feasible and sustainable. While significant progress has been made in the synthesis of pyridines, pyrazoles, indoles, and related frameworks, this methodology can in principle be extended to a broader range of *N*-heterocycles, including quinazolines, imidazoles, benzimidazoles, triazoles, and fused polycyclic systems. However, the applicability of microwave irradiation is not universal and strongly depends on the polarity of the solvents, the dielectric properties of the reagents, and the stability of the intermediates formed under high-energy conditions. Reactions involving non-polar substrates or thermally labile functionalities may be less amenable to microwave activation and scale-up can pose additional challenges due to limitations in microwave penetration depth. Thus, although microwave-assisted methodologies offer a versatile platform for *N*-heterocycle synthesis, careful consideration of substrate compatibility, reaction conditions, and scalability is required to fully exploit their potential. Clearly, microwave irradiation often leads to significantly reduced reaction times, in some cases decreasing from several hours to only a few minutes, which is attributed to rapid and uniform dielectric heating. Yields are frequently reported to be comparable or higher than those obtained under traditional conditions, particularly in reactions that benefit from localized superheating and enhanced molecular mobility. Selectivity outcomes, however, appear to be more context-dependent; while certain transformations demonstrate improved regio- or stereo-selectivity under microwave conditions, others show no marked difference compared to classical heating. These variations may arise from differences in the thermal profiles of the systems and the possible involvement of non-thermal microwave effects, although the latter remains a subject of ongoing debate. Overall, the emerging consensus is that microwave-assisted synthesis provides a powerful tool for improving efficiency and sustainability in organic chemistry, but more systematic studies directly contrasting it with conventional approaches are required to clarify its full advantages and limitations.

In applications of microwave reactions, solvents and catalysts play a crucial role in determining the efficiency and selectivity of MAOS, particularly in the synthesis of *N*-heterocycles. The choice of solvent is especially important, as its dielectric properties govern the extent to which microwave energy is absorbed and converted into heat. Polar solvents such as ethanol, methanol, or dimethylformamide (DMF) are generally more effective under microwave conditions because they couple efficiently with the electromagnetic field, leading to rapid and homogeneous heating. In contrast, non-polar solvents absorb microwave energy poorly, which may reduce the reaction rate or necessitate the use of co-solvents. Catalysts, both homogeneous and heterogeneous, further enhance the outcome of microwave-assisted transformations by lowering activation barriers and enabling milder conditions, often leading to higher yields and improved regio- or chemo-selectivity. Additionally, solid-supported catalysts and ionic liquids can act as microwave absorbers, creating localized “hot spots” that promote reaction efficiency. The synergistic interplay between solvent and catalyst selection therefore not only dictates the feasibility of microwave-assisted synthesis but also strongly influences product distribution, reaction kinetics and overall sustainability of the process.

### 2.1. Synthesis of N-Heterocycles

**Scheme 1:**
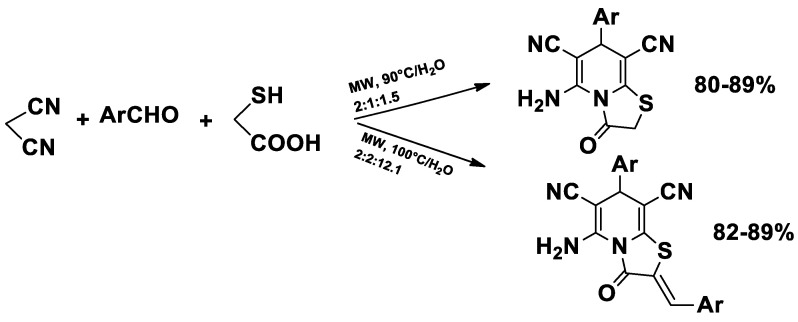
Synthesis of thiazolopyridines.

Thiazolopyridines and related fused heterocyclic compounds exhibit significant biological activity [49], and their chemical synthesis has been achieved via microwave-assisted reactions using malononitrile, aromatic aldehydes, and 2-mercaptoacetic acid in water (Scheme 1) [50].

These compounds exhibit several pharmacological activities, including antimicrobial, anticancer, anti-inflammatory and antiviral effects, making microwave-assisted strategies particularly valuable for rapidly generating libraries of bioactive derivatives for drug discovery.

**Scheme 2:**
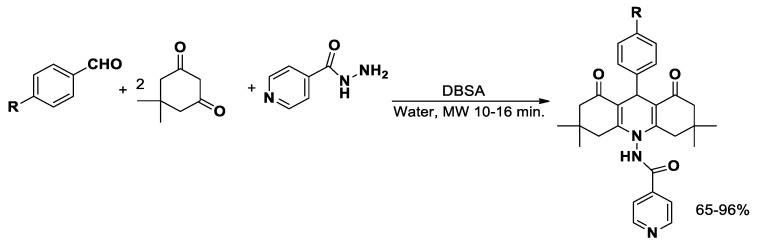
Synthesis of isoniazid analogues.

*p*-Dodecylbenzenesulfonic acid (DBSA) is an acidic catalyst with surfactant properties that facilitate the dissolution of organic materials. It is widely used to synthesize novel isoniazid (INH) analogues [51] via microwave irradiation using benzaldehydes and dimedone in water as reagents with 80–90% of yields (Scheme 2). Isoniazid analogues, derivatives of the well-known antitubercular agent, have been extensively investigated for their enhanced activity against *Mycobacterium tuberculosis*, including drug-resistant strains. In addition to their primary anti-tubercular effects, several analogues exhibit broader antibacterial properties, and some have demonstrated anticancer and enzyme inhibitory activities, highlighting their potential as versatile bioactive compounds. MAOS has proven particularly useful in the rapid and efficient generation of diverse isoniazid derivatives, facilitating the exploration of structure–activity relationships and the development of novel therapeutic candidates [52].

**Scheme 3:**
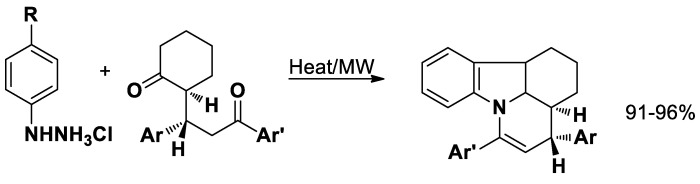
Synthesis of pyridocarbazoles.

The reaction between 2-(3-oxo-1,3-diarylpropyl)-1-cyclohexanones and phenylhydrazine hydrochloride in water results in the formation of pyridocarbazoles with excellent yield (Scheme 3) [53].

**Scheme 4:**
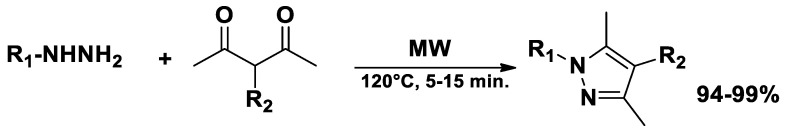
Synthesis of pyrazoles.

Pyrazoles and diazepines have been successfully synthesized under solvent- and catalyst-free conditions through microwave-assisted chemical synthesis, [54] achieving complete conversion at 120 °C in 5−15 min with 90% of yield (Scheme 4).

### 2.2. Cross-Coupling Reactions for Modifying Heterocycles

**Scheme 5:**
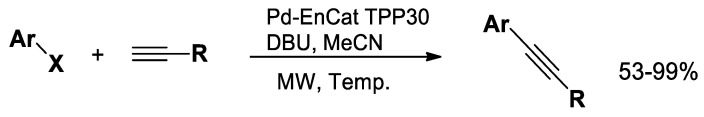
Cross−coupling reaction.

Additionally, C−C coupling reactions, such as the Sonogashira cross-coupling reaction, can be efficiently conducted under microwave irradiation [55,56,57]. Cross-coupling reactions, such as Suzuki and Sonogashira, enable the rapid functionalization of heterocycles, providing access to derivatives with enhanced antimicrobial, anticancer, anti-inflammatory and antiviral activities. When combined with microwave-assisted synthesis, these reactions offer shorter reaction times, higher yields, and efficient generation of pharmacologically relevant heterocyclic libraries for drug discovery. In particular, the Sonogashira cross-coupling reaction is typically performed using aryl iodides and bromides with terminal alkynes, utilizing reusable Pd-EnCatTM TPP30 (encapsulated Pd with PPh_3_) catalysts to obtain excellent yields (Scheme 5) [58].

**Scheme 6:**
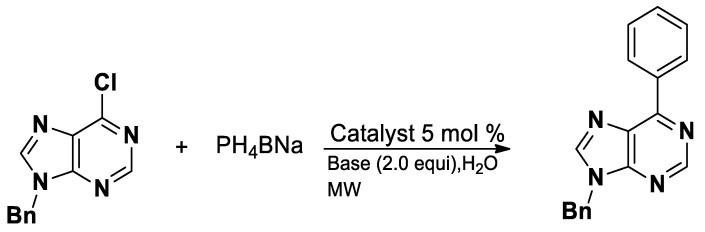
Synthesis of 6-arylpurines.

The synthesis of nucleosides from 6-chloropurines is enabled using an arylation reagent and sodium tetraarylborate in water under microwave irradiation (Scheme 6) [59].

These compounds demonstrated a wide range of pharmacological activities, including anticancer, antiviral and anti-inflammatory effects, often through modulation of kinases, nucleic acid synthesis, or enzyme inhibition. The introduction of diverse aryl groups at the C6 position enables fine-tuning of target specificity and potency, making 6-arylpurines valuable scaffolds in medicinal chemistry [60].

**Scheme 7:**
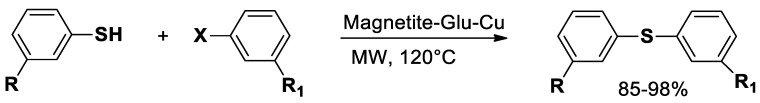
Coupling of thiophenols with aryl halides.

Furthermore, the formation of C−S bonds in several drug intermediates and pharmaceutical compounds (Scheme 7) [61] is achievable using magnetite-Glu-Cu as a catalyst for the reaction between aryl halide and thiophenol under microwave irradiation 90% of yield [55].

### 2.3. Click Chemistry: 1,3-Dipolar Cycloadditions

**Scheme 8:**
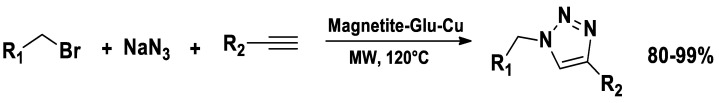
Synthesis of 1,2,3-triazoles.

In the case of click chemistry, microwave-assisted protocols have proven to be highly efficient at the laboratory scale; however, their translation to industrial settings requires careful attention to scalability and safety. Continuous-flow microwave reactors provide a practical solution, as they ensure controlled energy input, efficient heat dissipation and reproducible reaction outcomes while minimizing the risks associated with batch processing. Moreover, the inherently high selectivity and mild conditions of click reactions align well with green chemistry principles, facilitating safer operations and reducing environmental impact. Thus, the integration of advanced reactor technologies with microwave-assisted click chemistry offers a viable route toward sustainable large-scale applications. For instance, they can be utilized to perform 1,3-dipolar cycloadditions, commonly known as click chemistry, in which an alkyne reacts with an azide to form 1,2,3-triazoles. These triazoles can be selectively substituted at position four or five using magnetite-Glu-Cu as the reaction catalyst (Scheme 8). The underlying mechanism is generally attributed not to a change in the fundamental reaction pathway of the copper(I)-catalyzed azide–alkyne cycloaddition (CuAAC), but rather to the unique mode of energy transfer provided by microwave irradiation. Microwaves interact directly with polar molecules and ionic species in the reaction medium, leading to rapid and homogeneous volumetric heating that minimizes thermal gradients. This results in a higher effective concentration of reactive species at the catalytic sites and a reduction in activation energy barriers through efficient vibrational excitation. In contrast, traditional click chemistry relies on conventional convective and conductive heating, which is slower and often less uniform. Additionally, microwave irradiation has been proposed to promote catalyst activation and improve solubility of reagents, thereby increasing turnover frequency and selectivity. While no evidence supports a fundamentally different mechanistic pathway under microwave conditions, the enhanced kinetics and improved reaction environment provide significant advantages in terms of reaction efficiency, sustainability, and scalability.

The scope of microwave-assisted synthesis of 1,2,3-triazoles is extensive, offering a highly efficient and versatile approach for constructing these nitrogen-rich heterocycles. Microwave irradiation significantly accelerates the copper(I)-catalyzed azide–alkyne cycloaddition and other click chemistry reactions, often reducing reaction times from hours to minutes while improving yields and selectivity. This methodology has been successfully applied to a wide range of substrates, including aromatic, aliphatic, and functionalized azides and alkynes, enabling the rapid generation of diverse 1,2,3-triazole libraries for applications in medicinal chemistry, materials science, and chemical biology. Moreover, microwave-assisted protocols frequently allow solvent-free or green chemistry conditions, enhancing sustainability and operational simplicity. However, several limitations persist. The need for specialized microwave reactors can pose an accessibility barrier, and scale-up of microwave reactions remains challenging due to issues with uniform energy distribution in larger reaction volumes. Additionally, some sensitive functional groups may undergo degradation under high-energy microwave irradiation, and precise control over temperature and pressure is essential to avoid side reactions. Despite these challenges, microwave-assisted click chemistry remains a powerful and rapidly evolving tool in modern synthetic chemistry [62,63,64,65].

### 2.4. Cross and Ring-Closing Metathesis

A significant application of microwave reactions is in facilitating cross and ring-closing metathesis with high product yields using appropriate catalysts. For cross and ring-closing metathesis, microwave-assisted protocols have demonstrated significant advantages in terms of reaction rate and selectivity at the laboratory scale, yet their scalability and safety must be carefully addressed for industrial adoption. The use of continuous-flow microwave reactors allows for efficient temperature control, uniform energy distribution and improved catalyst performance, while also reducing the risks associated with pressure build-up in batch systems. In addition, the development of robust catalysts, solvent minimization, and strict adherence to safety protocols contribute to safer operation and lower environmental impact, thus enabling the sustainable large-scale application of microwave-assisted metathesis reactions. Metathesis is a specialized chemical reaction involving several olefins. Before discussing its microwave applications, it is essential to briefly analyze the catalysts developed for these reactions.

**Figure 5:**
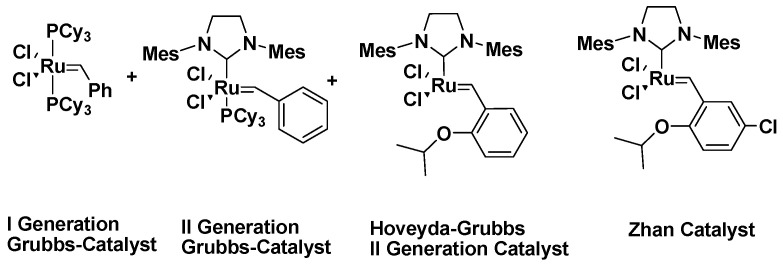
Ruthenium-based catalysts.

Schrock was the first to synthesize metathesis catalysts, producing a molybdenum-based catalyst that was unstable in air and required a nitrogen atmosphere for reactions. Grubbs later developed a ruthenium-based catalyst that was air-stable and yielded high product efficiency. The first-generation Grubbs catalyst contained two phosphine groups but exhibited low reactivity with polar-functionalized olefins. To address this, the second-generation catalyst replaced one phosphine group with an imidazolidinylidene core, which increased the electron density on ruthenium and improved the reactivity with polar functional groups. Further improvements led to the Hoveyda catalyst, a phosphine-free variant with an ortho-isopropoxy group that protected ruthenium from oxidative degradation during storage (Figure 5) [66,67].

Metathesis catalysts, particularly those based on ruthenium and molybdenum, exhibit distinct reactivity profiles and practical considerations that influence their selection for specific transformations. Ruthenium-based catalysts, such as the Grubbs first- and second-generation catalysts, are highly tolerant of functional groups, air, and moisture, making them versatile and user-friendly for a wide range of substrates in both laboratory and industrial settings. However, they generally exhibit lower activity in challenging sterically hindered or electron-deficient olefins compared to molybdenum-based catalysts. In contrast, molybdenum and tungsten alkylidene catalysts often demonstrate higher reactivity and selectivity, particularly in stereoselective and ring-closing metathesis reactions, but they are highly sensitive to air, moisture, and certain functional groups, requiring strictly inert conditions and careful handling. Newer ruthenium catalysts, including Hoveyda-Grubbs derivatives, attempt to balance these traits by combining enhanced stability with improved reactivity, though cost and catalyst loading remain considerations. Overall, the choice of catalyst involves a trade-off between stability, functional group tolerance, activity, and stereoselectivity, highlighting the importance of tailoring catalyst selection to the specific substrate and reaction conditions [68,69,70].

Microwave irradiation offers several distinct advantages in metathesis reactions compared to conventional thermal heating, primarily through rapid and uniform energy transfer that accelerates reaction rates. Enhanced heating efficiency allows reactions to reach the desired temperature in a fraction of the time required by traditional oil-bath or convective heating, often leading to significantly reduced reaction times and improved product yields. Additionally, microwave-assisted metathesis can promote more selective transformations by minimizing thermal gradients and localized overheating, which are common in conventional methods, thereby reducing side reactions and decomposition of sensitive substrates. The technique also facilitates solvent-free or green chemistry approaches, as polar solvents efficiently absorb microwave energy, further increasing reaction efficiency. However, while microwave irradiation provides clear operational advantages, its application is sometimes limited by the scale of the reaction, as uniform microwave penetration in larger volumes can be challenging, and specialized equipment is required. Overall, microwave-assisted metathesis represents a powerful tool for accelerating synthetic protocols and enhancing reaction efficiency while maintaining high selectivity, particularly in small-scale or laboratory settings.

Microwave-assisted reactions between allylbenzene (2.5 equiv.), olefins, and ruthenium catalysts II generation Grubbs catalyst, Hoveyda-Grubbs II generation catalyst (HGII), or Zhan Catalyst (2.5 mol%) were conducted under argon in refluxing dry CH_2_Cl_2_ for 5–12 h. The use of microwave irradiation significantly accelerated the reaction, promoting both cross-metathesis (CM) and abb, yielding predominantly E-isomers (99%) (Table 4) [66,71].

Under conventional conditions (batch synthesis), these reactions typically require 12 h with a heating mantle and reflux condenser. However, microwave reactors reduce reaction time substantially, making them more efficient.

Microwave applications in metathesis have been extensively studied. Literature reports confirm that microwave-assisted synthesis offers significantly higher yields than thermal heating in an oil bath. Several examples of batch and continuous-flow microwave organic synthesis have been explored, including esterification, aromatic nucleophilic substitution, and Claisen rearrangement, optimized under high-pressure and high-temperature conditions [72].

### 2.5. Impact of High Temperature and Pressure on Homogeneous Organic Synthesis

**Scheme 9:**
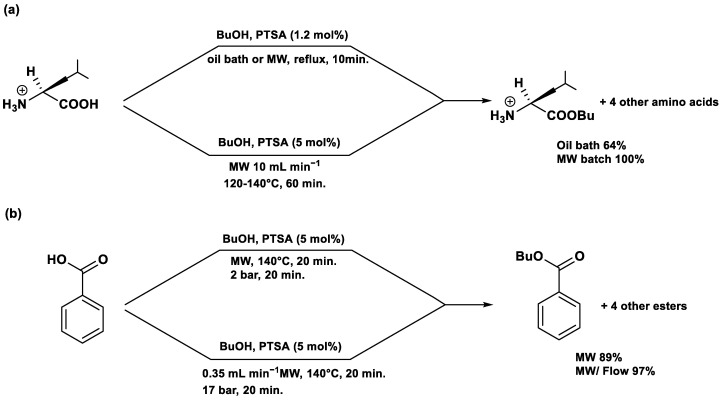
Fischer esterification reaction of (**a**) Leucine; (**b**) Benzoic acid.

For instance, the Fischer esterification of leucine with p-toluenesulfonic acid (PTSA) and butanol was successfully performed in a microwave reactor [73] (Scheme 9a). Comparing Fischer esterification in a round-bottom flask, tubular reactors of varying sizes, and a continuous-flow microwave reactor, it was observed that complete conversion was achieved under microwave conditions at reflux with a thin tubular reactor heated to 120−140 °C. In contrast, conventional batch-mode esterification achieved only 64% of the yield.

Additionally, benzoic acid esterification under microwave irradiation was optimized at 140 °C in batch mode, achieving >95% yield (Scheme 9b). The efficiency of microwave-assisted esterification, compared to traditional methods, highlights its effectiveness [74,75].

**Scheme 10:**
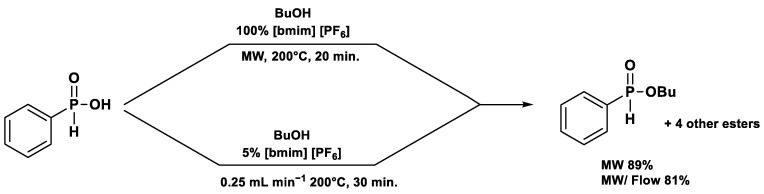
Organophosphorus transformations.

Furthermore, organophosphorus transformations using MW-assisted protocols are very interesting, particularly the esterification of phenyl-H-phosphinic acid, which benefits from the high temperature, such as in the alcoholysis of dimethyl H-phosphonate [76,77,78]. The reaction proceeded with the addition of [bmim][PF6], which enhances MW irradiation, whereas transesterification generally did not require a catalyst (Scheme 10).

**Scheme 11:**
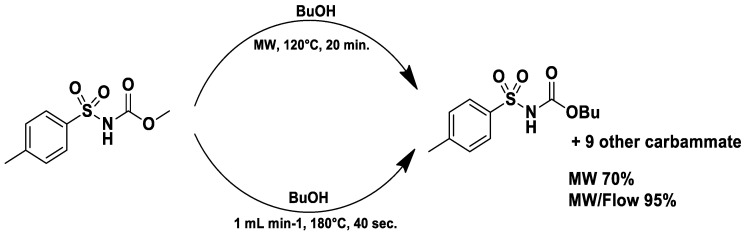
Transcarbamylation/transesterification of sulfonyl carbamate.

Other examples include the transcarbamylation/transesterification of sulfonyl carbamate using MW procedures performed at the solvent’s boiling point. The reaction time was reduced from 20 min to 40 s when butanol was heated from 120 °C in MW batch mode to 180 °C in MW flow mode with 70% of yield in MW and 95% MW/Flow (Scheme 11) [79,80].

**Scheme 12:**
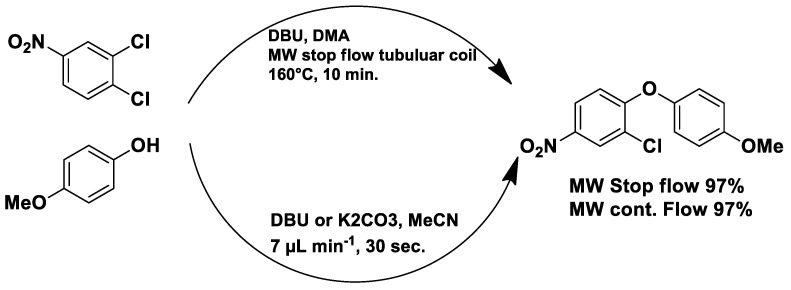
Scaling up of MW-assisted SNAr.

The SNAr reaction has been widely carried out using MW irradiation, and the procedure can be adapted to a stop-flow continuous MW protocol with 97% of yield [81]. The reaction was developed between 1,2-dichloro-4-nitrobenzene and 4-methoxyphenol in an 80 mL reaction vessel in the presence of an organic base (DBU) in DMA (Scheme 12).

**Scheme 13:**
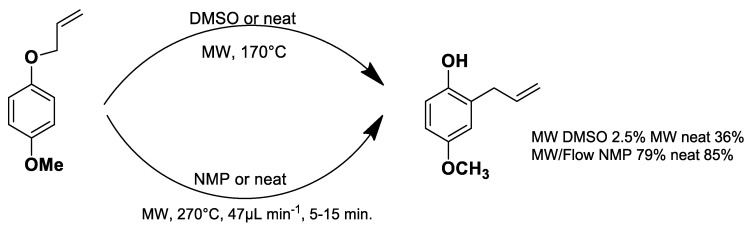
Claisen rearrangement.

Claisen rearrangement is promoted under high-temperature and high-pressure conditions, and MW irradiation has been exploited to enhance reaction conversion in continuous MW synthesis. The conversion of 1-allyloxy-4-methoxybenzene in N-methyl-2-pyrrolidone (NMP) at 270 °C (Scheme 13) [82] resulted in 78–85% yield within 5 to 15 min. A two-fold yield improvement was observed compared to the reaction heated in an oil bath [83].

**Scheme 14:**
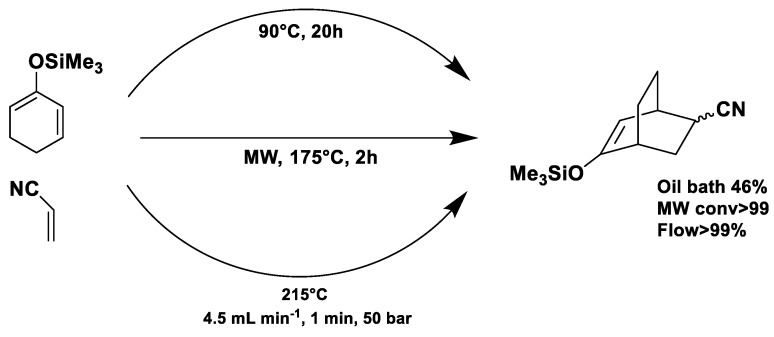
MW-assisted Diels–Alder reaction.

Other examples include the Diels–Alder reaction under MW irradiation and flow mode. MW irradiation was used to optimize the protocol before applying it to conventional flow mode. The reaction between (cyclohexa-1,5-dien-1-yloxy) trimethylsilane and acrylonitrile occurred under MW irradiation, where polymerization of acrylonitrile was significantly reduced compared to conventional oil bath heating (Scheme 14) [84].

Since acrylonitrile polymerization causes microreactor clogging, MW optimization was performed at different temperatures up to 175 °C. It was observed that at 175 °C, acrylonitrile was completely consumed within 2 h. The procedure was then adapted to flow mode, increasing the reaction temperature to 30 °C and decreasing the reaction time to 2 min. Conventional continuous flow was performed at 215 °C with a residence time of 60 s, yielding final compound in 45 min with a 95% recovery.

**Scheme 15:**
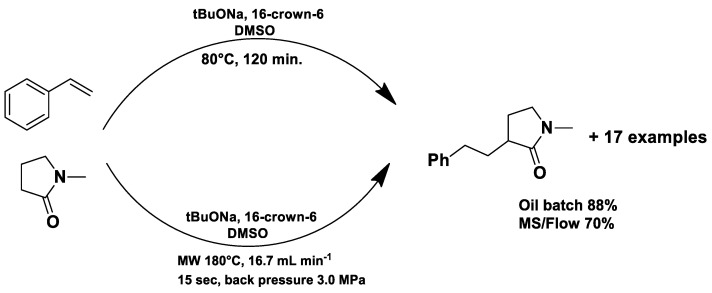
*C*-Alkylation of *N*-alkylamides.

Another application involves the C-alkylation of *N*-alkylamide by microwave irradiation, which was scaled up to MW continuous flow mode (Scheme 15) [85]. The reaction was performed between styrene and dimethylacetamide using t-BuOK as a base, and excellent results were obtained when the procedure was translated into flow mode.

### 2.6. Applied Homogeneous and Heterogeneous Catalysis in MW

**Scheme 16:**
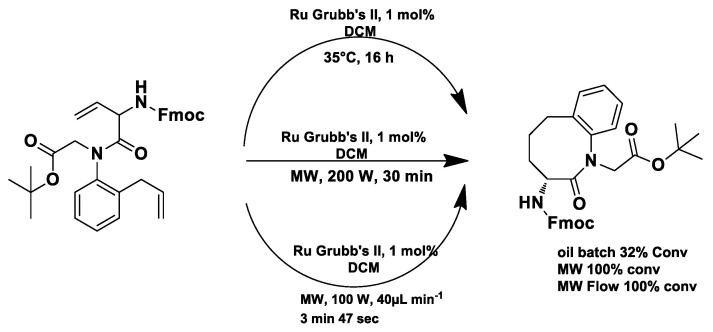
Ruthenium-catalyzed ring-closing metathesis.

MW-assisted synthesis has been widely applied to organometallic reactions [86,87,88,89]. Ru-catalyzed metathesis has been reported under MW irradiation [90,91,92] including an example of ring-closing metathesis (RCM) [92], using the II-generation Grubbs catalyst for the synthesis of macrocycle. A comparison between conventional conditions, MW batch irradiation, and continuous flow irradiation with a capillary-based flow system (Scheme 16) showed that MW irradiation led to higher conversion rates, suggesting a direct effect on the reagent and catalyst. As demonstrated, temperature expectations can be surpassed when MW irradiation is used with polar substrates in non-absorbing solvents [93]. MW energy can thus be considered an important variable for achieving selectivity in organic synthesis [94].

**Scheme 17:**
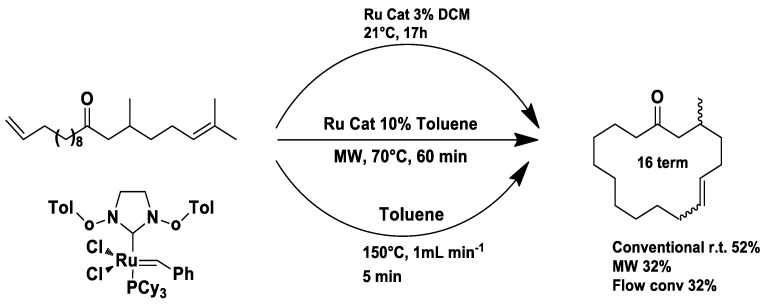
Macrocyclization.

The synthesis of a macrocycle under MW and flow conditions was carried out in a microwave reactor, where it was observed that at high temperatures, the catalyst decomposed. Additionally, the flow protocol trapped the ethylene produced during the reduction (Scheme 17) [95]. Equivalent results were obtained in the synthesis of a C-16 macrocycle at 70 °C with high yields.

MW irradiation has also been widely applied to the preparation of metallic nanoparticles and small nanocatalysts [96,97,98,99]. These molecules exhibit direct pharmacological activities, including antimicrobial, antiviral, anti-inflammatory, and anticancer effects. These activities are often attributed to their high surface area, tunable size, and surface chemistry, which enable interactions with microbial membranes, viral particles, or cellular components. In addition, metallic nanocatalysts can facilitate the rapid and efficient synthesis of bioactive molecules under mild conditions, combining synthetic utility with potential therapeutic relevance [100].

**Scheme 18:**
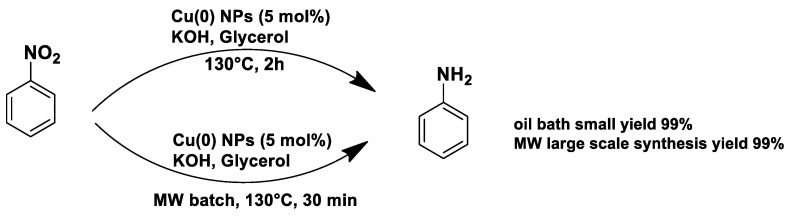
Reduction of nitrosobenzene.

The effects of MW irradiation on solid-supported catalysts are well recognized [101,102,103,104] as the impact of flow chemistry on process intensification [105]. Several innovative protocols in catalytic oxidation and reduction have been extensively studied under MW and flow chemistry. For example, the copper-catalyzed reduction of nitrobenzene in glycerol (Scheme 18) [106] achieved a 95% yield.

**Scheme 19:**
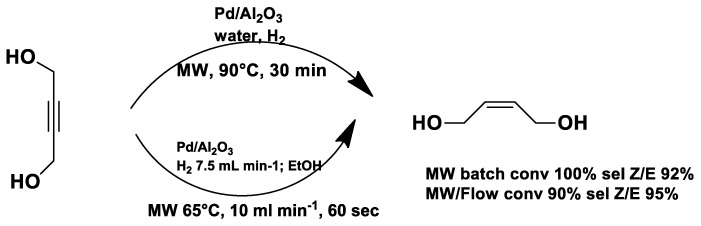
Semyhydrogenation of 1,4-butynediol in MW batch mode and continuous mode.

In addition, the semi-hydrogenation of 1,4-butynediol was performed [107] in MW batch mode and continuous flow mode with a controlled flow of 7.5 mL of H_2_, aiming to achieve high product yield and selectivity toward cis-2-butene-1,4-diol using a selective palladium nanoparticle catalyst on an aluminum sphere (Scheme 19).

**Scheme 20:**
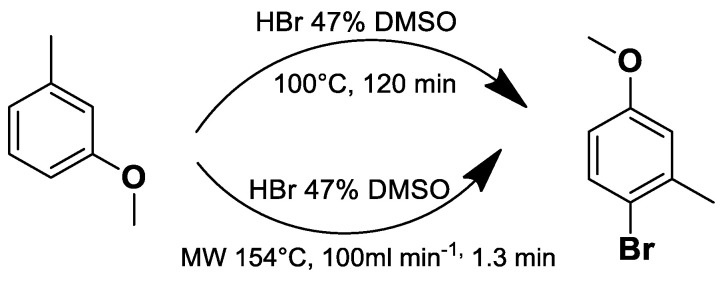
Oxidative bromination of 3-methyl anisole.

MW and continuous flow methods also allow for aryl bromide synthesis [108,109] (Scheme 20). The reaction produced 4-bromo-3-methylanisole with a retention time of just 1.3 min, achieving a yield of 79% and producing 0.32 kg of the product in 5 h.

**Scheme 21:**
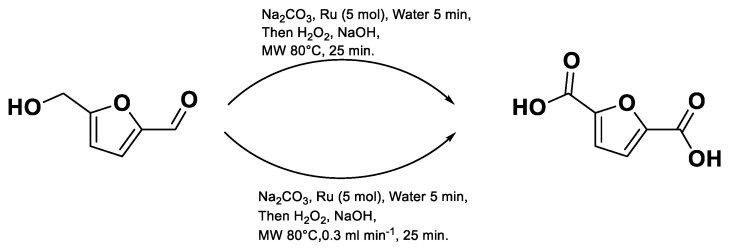
Ruthenium-catalyzed oxidation of HMF.

MW irradiation has been widely used in glycosylation reactions [110,111]. A continuous step was studied for the preparation of adenosine via glycosylation and deprotection [112]. Another example involves the catalytic oxidation of 5-hydroxymethylfurfural (HMF) [113] using aqueous H_2_O_2_ and air, employing Ru/C catalysis under MW and continuous-flow MW irradiation (Scheme 21) [114].

**Scheme 22:**
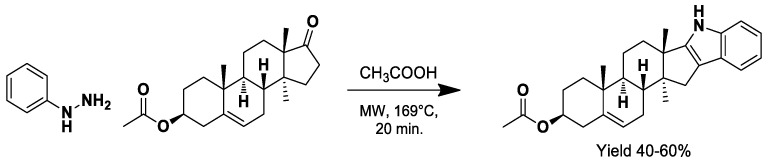
MW-assisted Fisher indole synthesis.

The synthesis of heterocycles via MW had a strong environmentally sustainable impact [115,116] on medicinal chemistry, polymer synthesis, and material science. Many green solvents and catalyst-free protocols have been explored. One example is the MW-assisted Fischer indole synthesis, which has been proposed in MW batch mode using homogeneous [117] or heterogeneous acidic catalysts [118] and later scaled up to MW continuous mode [119] Starting from dehydroepiandrosterone acetate and phenylhydrazine hydrochloride in glacial acetic acid (Scheme 22), the product was obtained with a 40–60% yield [120].

Clearly, microwave-assisted Fischer indole synthesis is a powerful and efficient strategy for the construction of indole scaffolds, which are prevalent in numerous biologically active molecules and pharmaceuticals. Indoles synthesized via this method exhibit a wide range of pharmacological activities, including anticancer, antimicrobial, antiviral, anti-inflammatory and central nervous system (CNS) modulatory effects. The use of microwave irradiation significantly reduces reaction times, improves yields, and often enhances regio- and chemo-selectivity compared to conventional heating, enabling the rapid generation of diverse indole derivatives for medicinal chemistry and drug discovery [121].

### 2.7. Synthesis of Heterocycles and Multicomponent Reaction

**Scheme 23:**
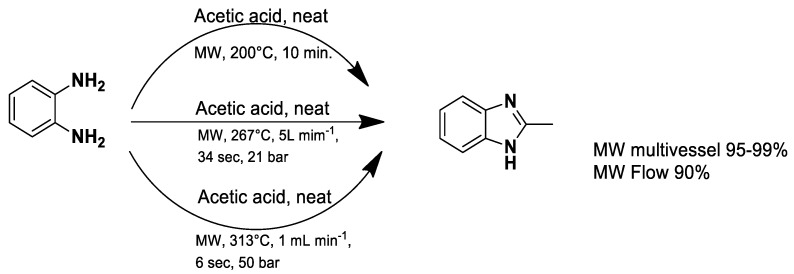
Synthesis of imidazole.

The synthesis of methyl benzimidazole was performed using o-phenylenediamine in acetic acid, a simple condensation procedure carried out in MW reactors (Scheme 23). The reaction was completed in 10 min with a yield of 90% [122].

These derivatives exhibit a wide range of biological activities, including antimicrobial, antiviral, anticancer, anti-inflammatory, and antihypertensive effects, often mediated through interactions with enzymes, DNA, or cellular receptors. The introduction of methyl groups at specific positions on the benzimidazole ring can modulate lipophilicity, target binding affinity and metabolic stability, thereby enhancing the therapeutic potential of these compounds. Methyl benzimidazoles have been employed as scaffolds in the development of antiparasitic agents, proton pump inhibitors and kinase inhibitors, highlighting their utility in medicinal chemistry [123].

**Scheme 24:**
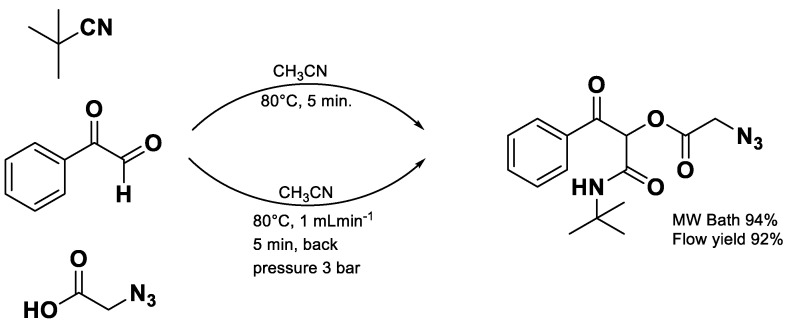
Isocyanide-based multicomponent Passerini reaction.

MW irradiation has been highly beneficial for the synthesis of heterocycles, given their wide applicability in pharmaceutical chemistry. MW irradiation reduces reaction times and enhances efficiency through multicomponent reactions. Numerous protocols have been reported in the literature, including the isocyanide-based multicomponent Passerini reaction via MW irradiation (Scheme 24) [124], yielding α-acyloxy ketone derivatives at 0.312 g/min productivity.

**Scheme 25:**
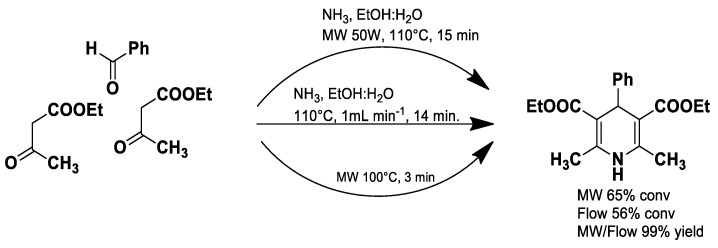
Hantzsch multicomponent synthesis.

Hantzsch synthesis has also been explored under MW irradiation [125,126]. For example, 4-aryl-1,4-dihydropyridines were synthesized, [127] and reaction conversion was compared with conventional flow mode (Scheme 25) [128]. The reaction under MW irradiation occurred at 140 °C for 10 min. In conventional flow mode [129], formaldehyde was used instead of benzaldehyde, and the yield was lower in flow mode (68%) compared to MW batch mode (82%).

**Scheme 26:**
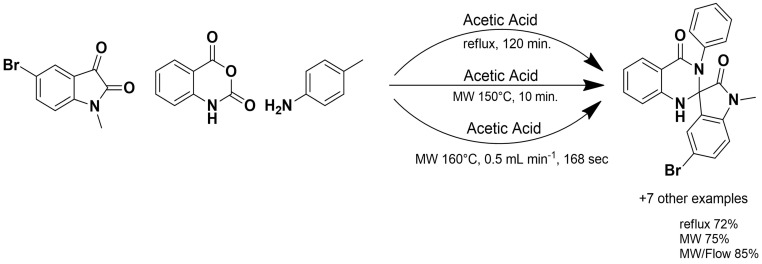
Three-component spiro-oxindole synthesis.

Three-component spiro-oxindole synthesis has been studied in the literature (Scheme 26) [130]. The combination of MW irradiation with continuous flow in a silicon carbide tubular reactor improved the yield [131].

The spiro-oxindole core is frequently found in natural products and synthetic analogues with potent activity against targets such as kinases, proteases and bacterial or viral enzymes. Modifications at the spiro center and adjacent positions enable fine-tuning of potency, selectivity, and pharmacokinetic properties, making spiro-oxindoles valuable scaffolds in medicinal chemistry. In this context, multicomponent reactions (MCRs) of spiro-oxindole class have transformed modern organic synthesis. In contrast to conventional methods that generally involve the coupling of two reactive species, MCRs integrate three or more substrates in a single, one-pot process to generate the desired product [132,133].

This product typically incorporates most, if not all, of the atoms from the starting materials, thereby ensuring high efficiency and excellent atom economy. MCRs proceed through a sequence of mechanistic steps in which distinct functional groups react in a defined and orderly manner [134,135].

In addition to their pharmacological relevance, spiro heterocycles hold significant potential in materials science. Their unique structural frameworks and electronic characteristics render them attractive candidates for applications in organic semiconductors, light-emitting diodes, and liquid crystalline materials [136,137,138,139].

Notably, spiro-oxindoles exhibit a broad spectrum of biological and pharmacological activities. These include anxiolytic, antibacterial [140,141,142], antitubercular [143], antioxidant [144], antitumor [145], antiprotozoal [146], anti-inflammatory [147], anticonvulsant [148], anthelmintic [149], antiepileptic [150], antimycobacterial [151], antimalarial [152], anticancer activities [153,154,155,156,157], and antileishmanial [158].

**Scheme 27:**
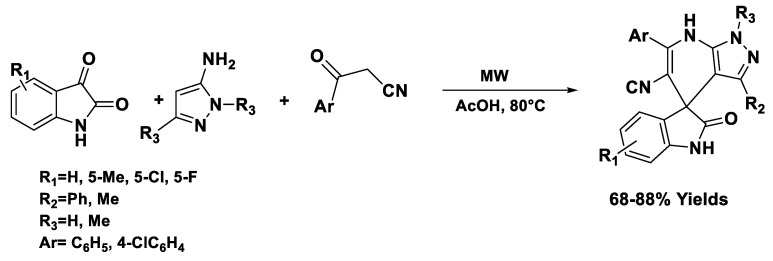
Synthesis of tetrahydrobenzo[b]pyran derivatives.

Tu and co-workers established a three-component microwave-assisted protocol for the synthesis of novel spiro[indoline-3,4′-pyrazolo[3,4-b] pyridine] derivatives. The target spiro frameworks were efficiently constructed from readily available isatins, pyrazol-5-amines, and ketonitriles. As illustrated in Scheme 27, acetic acid (HOAc) served a dual function as both the reaction medium and an acid catalyst. The reaction proceeded smoothly under microwave irradiation at 80 °C. This approach offers a versatile and efficient route for compound library generation in drug discovery, owing to its operational simplicity, broad functional group tolerance, and mild reaction conditions [159].

**Scheme 28:**
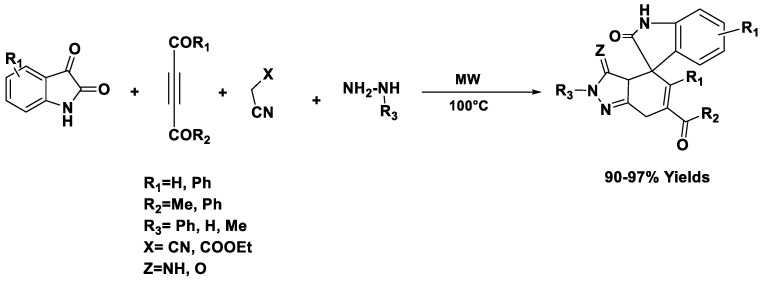
Synthesis of pyrano[2,3-d]pyrimidine, pyrano[3,2-b]pyran, and chromeno[2,3-d]pyrimidine derivatives.

Furthermore, Ali and co-workers developed a rapid and efficient microwave-assisted, solvent-free methodology for the synthesis of polyfunctionalized spiro[indoline-3,4′-pyrazolo[3,4-b]pyridines] exhibiting notable pharmacological potential (Scheme 28). Utilizing a straightforward and highly effective multicomponent reaction (MCR) strategy involving hydrazines, dimethyl acetylenedicarboxylates (DMADs), isatin, and active methylene compounds, the desired products were obtained in excellent yields [160].

**Scheme 29:**
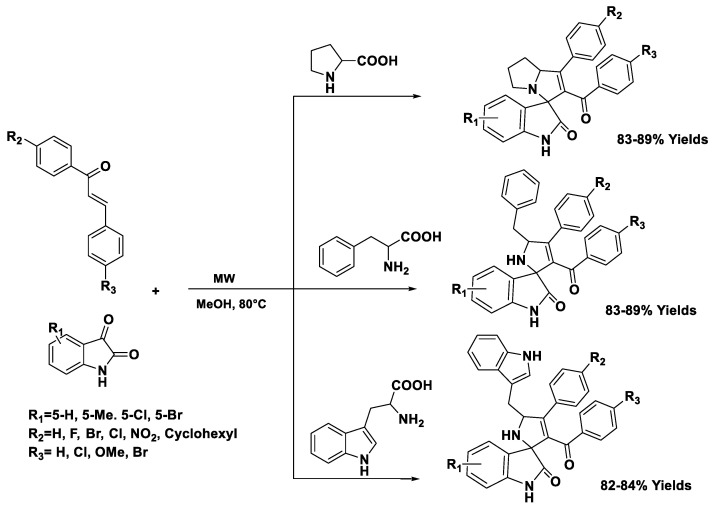
Synthesis of functionalized spiro[indoline-3,20-pyrrolidin]-2-one/spiro[indoline-3,30-pyrrolizin]-2-ones.

The antimicrobial properties of the synthesized compound were assessed against *Staphylococcus aureus* (Gram-positive), *Pseudomonas aeruginosa* (Gram-negative), *Candida albicans* (yeast), and *Aspergillus niger* (fungus) using the agar diffusion method. Most compounds displayed moderate to strong antimicrobial activity. Additionally, the total antioxidant capacity of each compound was determined using the phosphomolybdenum assay. Cytotoxic evaluation against three human cancer cell lines HePG2, CT-116, and MCF-7 revealed cytotoxic effects ranging from weak to potent. Leishmaniasis is a neglected tropical disease, and its treatment urgently requires improvement. To address this need, Coghi et al. developed a green, three-component reaction for the synthesis of novel compounds with antileishmanial properties. A new series of functionalized spiro[indoline-3,2′-pyrrolidin]-2-ones and spiro[indoline-3,3′-pyrrolizin]-2-ones were synthesized from natural product-inspired, pharmaceutically privileged bioactive substructures, namely, isatins, various substituted chalcones, and amino acids via 1,3-dipolar cycloaddition reactions in methanol at 80 °C under microwave-assisted conditions, affording good to excellent yields (Scheme 29) [161].

We reported in vitro antileishmanial activity and structure–activity relationship (SAR) studies against *Leishmania donovani*. The compounds proved to be highly active, with IC_50_ values of 2.43 μM, 0.96 μM, 1.62 μM, and 3.55 μM, compared to Amphotericin B (IC_50_ = 0.060 μM). Furthermore, relative to the standard drug Camptothecin, all compounds were evaluated for their ability to inhibit *Leishmania* DNA topoisomerase type IB.

**Scheme 30:**
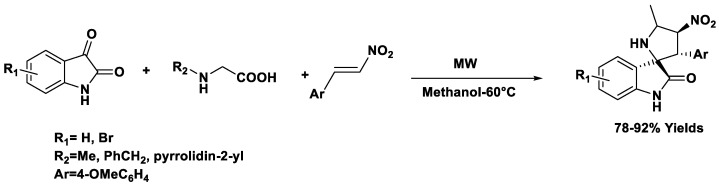
Synthesis of nitrostyrene-based spiro-oxindole derivatives.

In 2023, Wong and co-workers reported a highly efficient microwave-assisted multicomponent reaction (MCR) for the synthesis of a series of nitrostyrene-based spiro-oxindoles with excellent chemo- and regioselectivity via a [3 + 2] cycloaddition (Scheme 30). In this Huisgen-type reaction, readily available substituted isatins, various α-amino acids, and (E)-2-aryl-1-nitroethenes were subjected to microwave irradiation at 300 W in methanol, affording the desired spiro adducts in good-to-high yields [162].

The stereochemical configuration of the pyrrolidine ring in the spiro[pyrrolidine-2,3′-oxindole] derivatives were confirmed by single-crystal X-ray diffraction analysis. The synthesized compounds exhibited notable anticancer potential when evaluated against human lung cancer (A549) and human liver cancer (HepG2) cell lines, as well as immortalized normal lung and liver cell lines. Among them, five derivatives displayed significant in vitro anticancer activity (IC_50_ = 34.99–47.92 μM; SI = 0.96–2.43) against the A549 cell line, while six compounds demonstrated promising activity against HepG2 cells. Notably, these compounds showed higher potency and selectivity compared with the standard reference artemisinin (IC_50_ = 100 μM; SI = 0.03) in the A549 assay. However, none of the tested compounds exhibited substantial cytotoxic effects against both lung (A549) and liver (HepG2) cancer cells.

It is possible to conduct multicomponent reactions (MCRs) under solvent-free and/or catalyst-free conditions using a microwave (MW). This strategy irradiation represents a particularly sustainable and efficient tool in organic synthesis. In the absence of solvents, reactants are in close contact, resulting in higher effective concentrations and improved reaction rates, while omitting catalysts eliminates the need for additional purification steps and avoids metal contamination key advantages for both pharmaceutical and materials-oriented synthesis. The synergistic effect of MW irradiation and neat reaction conditions often leads to shorter reaction times, higher yields, and greater selectivity, in full accordance with the principles of green chemistry.

In such protocols, MW heating provides rapid and uniform energy input, often enabling enhanced reaction rates and selectivities compared to classical thermal methods [163]. Several studies have validated the remarkable potential of such systems. Yin et al. reported the solvent- and catalyst-free MW-assisted synthesis of hydroxylated 2,4,6-trisubstituted pyridines in excellent yields within minutes, demonstrating the efficiency of neat MCR conditions [164].

**Scheme 31:**
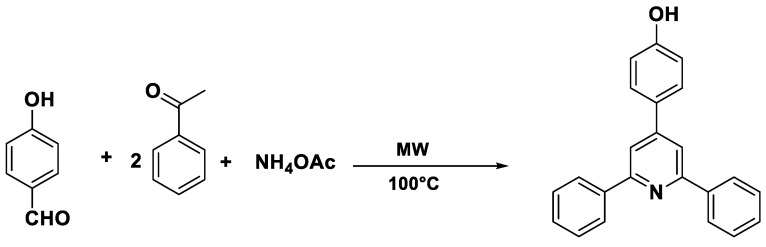
Synthesis of 4-(2,6-diphenylpyridin-4-yl) phenol via a solvent- and catalyst-free multicomponent reaction.

They investigated the synthesis of 4-(2,6-diphenylpyridin-4-yl) phenol through a three-component condensation involving 4-hydroxybenzaldehyde, acetophenone, and ammonium acetate to determine the optimal reaction parameters (Scheme 31). The reactions were performed under microwave-assisted conditions (400 W) in the absence of any solvent or catalyst, with reaction temperatures varied between 60 and 120 °C. Clearly, the elimination of solvents reduces waste streams and simplifies downstream processing, while the omission of catalysts avoids issues of metal contamination, catalyst recovery and deactivation. More recently, Kerru et al. demonstrated a catalyst-free MW-promoted four-component MCR in aqueous medium, achieving functionalized 1,4-dihydropyridine scaffolds in short reaction times and excellent yields [165].

### 2.8. Stereoselectivity in Microwave-Assisted Synthesis

**Scheme 32:**
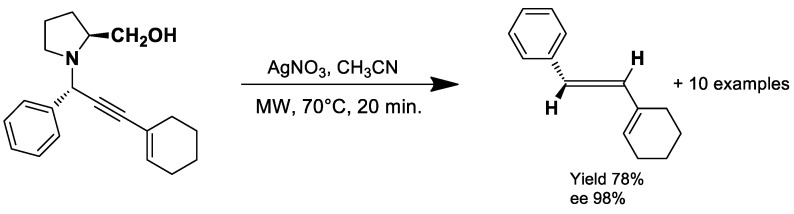
Stereoselective synthesis.

Stereoselective synthesis under MW irradiation has been widely reported. Selectivity is crucial in organic synthesis and MW heating offers a promising field for controlling regio- and stereo-selectivity [166]. Scheme 32 illustrates how vinyl allenes can be obtained enantioselectively from alkynes using AgNO_3_ in acetonitrile at 70 °C under MW irradiation, ref. [99] achieving an enantiomeric excess of 79–99%.

**Scheme 33:**
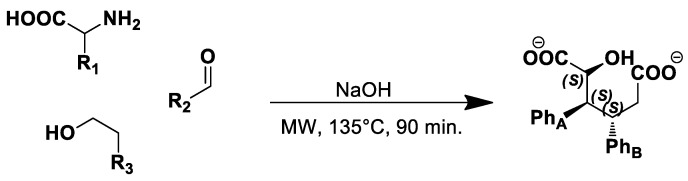
Multicomponent diastereoselective synthesis.

Another synthesis was carried out under MW irradiation, involving the condensation of L-phenylalanine and benzaldehyde in ethanol in alkaline conditions (Scheme 33) [167]. This reaction yielded a single crystalline compound with the structure of a dicarboxylic acid, which was isolated and characterized by X-ray crystallography as a racemic mixture of *R,R,R* and *S,S,S* enantiomers.

**Scheme 34:**
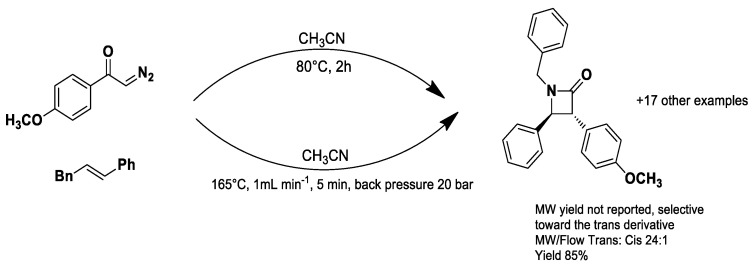
Wolff–Staudinger reaction of *β*-lactams.

The Wolf–Staudinger reaction was also carried out under MW irradiation, enabling the synthesis of *β*-lactams via [2 + 2] cycloaddition of ketenes with imines. Diastereoselectivity for trans-substituted reactions was studied in both MW batch mode [168] and continuous MW operation (Scheme 34) [169].

## 3. MW-Assisted Nanoparticles Synthesis

The evolution of MAOS techniques has significantly advanced the field of nanomaterials fabrication, offering rapid and efficient methods to produce nanoparticles with tailored properties for a wide array of applications. These methods leverage the unique characteristics of microwave irradiation to accelerate chemical reactions and enable the synthesis of nanomaterials under controlled conditions. Among the various MAS approaches, hydrothermal and solvothermal synthesis are prominent techniques that utilize high temperature and pressure to facilitate the nucleation and growth of nanoparticles in aqueous and organic solvents, respectively. These methods have been extensively studied for their ability to produce nanoparticles with uniform size and morphology, which are crucial for applications in catalysis, electronics, and energy storage [170].

In addition to hydrothermal and solvothermal methods, microwave energy is employed in several other nanomaterial synthesis approaches, including sonochemical synthesis, combustion synthesis, solid-state synthesis, and plasma-assisted synthesis. Sonochemical synthesis combines microwave irradiation with ultrasonic waves to induce chemical reactions in liquid media, resulting in the rapid formation of nanomaterials with enhanced yield and precise control over particle size and morphology [171].

Combustion synthesis, particularly microwave-heated combustion synthesis (MHCS), utilizes microwave heating to initiate exothermic reactions between metal precursors and fuel sources, producing nanomaterials with controlled stoichiometry and phase purity at accelerated reaction rates. However, MHCS is characterized by extremely rapid reactions, and issues regarding scalability and control over byproduct formation remain underexplored [172]. Microwave-assisted solid-state synthesis involves the use of microwave irradiation to trigger reactions between solid reactants or precursors at elevated temperatures, allowing for the formation of complex nanostructures via solid-state transformations. This method offers advantages such as solvent-free processing and energy efficiency, making it an attractive option for the synthesis of various nanomaterials [173].

Plasma-assisted synthesis employs microwave energy to generate and sustain plasma discharges, enabling fine control over nanoparticle size, shape, and surface characteristics. Despite its high energy requirements, plasma-assisted synthesis offers unique capabilities for the fabrication of nanomaterials with tailored properties, provided that renewable energy sources are integrated to mitigate sustainability concerns [174].

Collectively, these MAS techniques demonstrate high reaction efficiency, scalability, and the potential for producing high-quality nanomaterials suitable for applications in catalysis, electronics, energy storage, and biomedical engineering. The ability to control parameters such as microwave power, irradiation time, and precursor concentration allows for the precise tuning of nanoparticle properties to meet specific application requirements. Moreover, the rapid heating provided by MAS not only reduces reaction times but also enhances energy efficiency, aligning with green chemistry principles and reducing the environmental impact of nanoparticle synthesis [175]. Therefore, the continued development and optimization of MAS techniques hold significant promise for the sustainable and efficient synthesis of nanomaterials with tailored properties. By addressing existing challenges and integrating renewable energy sources, MAS can play a pivotal role in advancing the field of nanotechnology and its applications across various industries.

## 4. MW-Assisted Synthesis in Medicinal Chemistry

MAOS, when conducted in dedicated microwave reactors, has matured into a robust and widely utilized methodology in drug discovery, primarily due to its ability to accelerate reactions, enhance yields, improve product purity, and promote greener reaction conditions. In recent years, the application of microwave irradiation to heterocyclic synthesis has attracted significant attention, continuous technological advances have considerably expanded the scope and impact of this technique in synthetic and medicinal chemistry. Microwave irradiation (MW) emphasizes key operational details regarding the target *N*-heterocycle and its biological or pharmacological activity. Heterocyclic ring systems have garnered significant attention owing to their frequent presence in a wide range of biologically active molecules. A survey of prominent pharmacophores reveals that nitrogen-containing heterocycles constitute the most common structural motif among biologically relevant small molecules. To date, *N*-heterocycles continue to serve as versatile scaffolds for compounds exhibiting diverse biological activities and find extensive application across various pharmacological fields [176,177,178,179,180,181,182,183,184].

### 4.1. Antimicrobial Activity

**Scheme 35:**
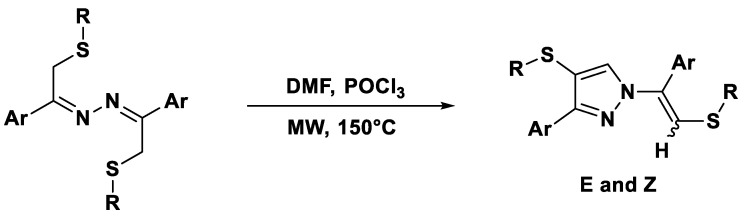
Synthesis of substituted pyrazoles.

One of the first compounds employed in the treatment of tuberculosis was pyridine derivative isoniazid, which was commonly administered in combination with other drugs such as ethambutol, rifampin, streptomycin, and pyrazinamide. However, the growing drug resistance among bacterial strains has necessitated the development of structural analogues or novel derivatives, many of which contain pyrazole moieties known for their potent biological activity and crucial roles in various biochemical systems [185]. Several microwave-assisted synthetic approaches have been reported for the preparation of such *N*-heterocycles. Muthusubramanian and co-workers developed a rapid microwave-assisted protocol for synthesizing arylthio- and cyclohexylthio-substituted pyrazoles (Scheme 35) [186].

The reaction of *u*-arylthio-substituted acetophenoneazines with Vilsmeier’s reagent at 0 °C resulted in complete conversion to the corresponding products. Cyclization was subsequently achieved under microwave irradiation at 150 °C for 30–60 s. The *E* isomer was obtained as the predominant product, although minor amounts of isomerization by-products were also detected in some cases. The antimycobacterial activity of the isomeric pyrazoles (*E* and *Z*) was evaluated, revealing that the *E* isomer exhibited superior activity. Moreover, the cyclohexylthio-substituted pyrazoles generally displayed higher potency than their arylthio analogues. The sequencing of the *Mycobacterium tuberculosis* (MTB) genome has yielded critical insights essential for the rational design of new drugs targeting this pathogen. Recent studies have indicated that interfering with MTB lipid metabolism pathways could represent an effective strategy for weakening or eradicating the bacterium. McLean and colleagues demonstrated that certain azole derivatives act as potent inhibitors of cell growth in *Mycobacterium bovis* and *Mycobacterium smegmatis*, two species that closely resemble MTB [187].

**Scheme 36:**
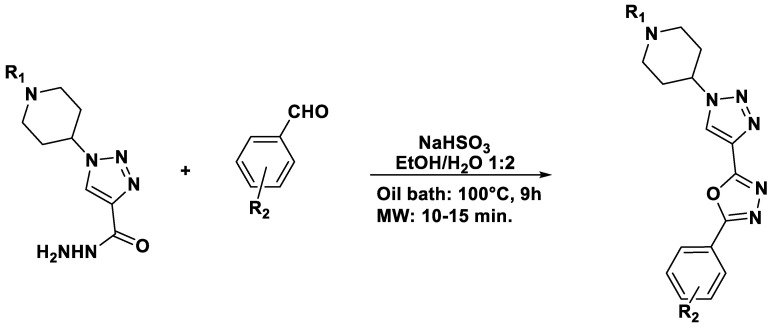
Synthesis of 1,3,4-oxadiazoles.

1,3,4-Oxadiazoles represent an important class of heterocyclic compounds and serve as core scaffolds in numerous bioactive molecules [188] 2,5-unsymmetrically disubstituted derivatives possess significant biological relevance [189]; however, their synthesis is often challenging. Fortunately, microwave (MW) irradiation can markedly enhance reaction rates and yields. Building upon their previous research, Shinde et al. developed a novel MW-assisted method for the synthesis of 2,5-disubstituted 1,3,4-oxadiazoles using a MicroSYNTH MW labstation and sodium bisulfite as a catalyst [190]. The reaction, carried out in ethanol by combining the hydrazide and aromatic aldehyde, afforded up to 95% yield within 10–15 min at 100 °C. In contrast, conventional heating required at least 9 h for complete cyclization (Scheme 36).

**Scheme 37:**
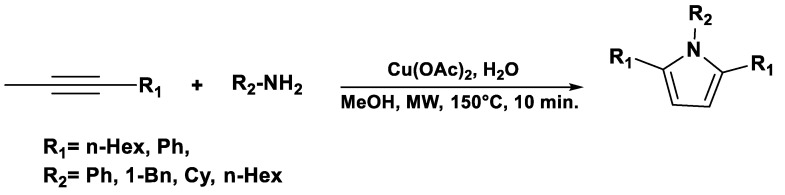
Synthesis of pyrrole scaffold.

All synthesized compounds were evaluated for their in vitro antifungal activity. It was found that substituents on the piperidine nitrogen and at position 5 of the oxadiazole ring significantly influenced biological activity. Derivatives bearing a methyl sulfone group on the piperidine nitrogen and a chlorine atom on the phenyl ring exhibited antifungal potency comparable to miconazole against *Fusarium oxysporum* and *Candida albicans*. Replacement of the chlorine atom with a hydroxyl group maintained similar activity, showing comparable efficacy to miconazole against *Candida albicans*, *Aspergillus flavus*, *Aspergillus niger*, and *Fusarium oxysporum*. Among all tested compounds, those featuring a sulfonyl group at R_1_ and either Cl or OH substituents at R_2_ were the most active. These groups can thus be regarded as key pharmacophores for the design and development of new antifungal agents. Lee et al. successfully synthesized pyrroles as potent antitubercular scaffold with a higher degree of substitution than previously reported examples through a Cu(II)-catalyzed, one-pot microwave (MW) irradiation reaction between 1-alkynes and primary amines, affording *N*-2,5-trisubstituted pyrroles (Scheme 37) [191].

The enhanced performance of this reaction under MW conditions can be directly attributed to the heterogeneous nature of the reaction system. It consists of a highly polar solvent, methanol which efficiently absorbs microwave energy and a ferromagnetic metal catalyst, copper, capable of strong interaction with the electromagnetic field. A “nonequilibrium local heating” effect has been observed in similar heterogeneous systems, where the reaction temperature is determined by both the dielectric loss of the polar solvent and the heat transfer from the ferromagnetic metal particles to the solvent. Under these optimized MW conditions, the reaction afforded alkyl- and aryl-substituted pyrroles in good yields (42–82%) within short reaction times (<10 min) [192]. The pyrrole ring, whether incorporated as a substituent or bearing various modifications on the ring itself, is one of the most observed heterocyclic systems in natural products and synthetic compounds. This prevalence is likely due to the electronic properties of the pyrrole ring, which enhance binding interactions with enzymes and receptors and allow further scaffold modifications to optimize biological activity. Pyrroles exhibit notable medicinal applications, including pronounced antimycobacterial activity [193,194].

**Figure 6:**
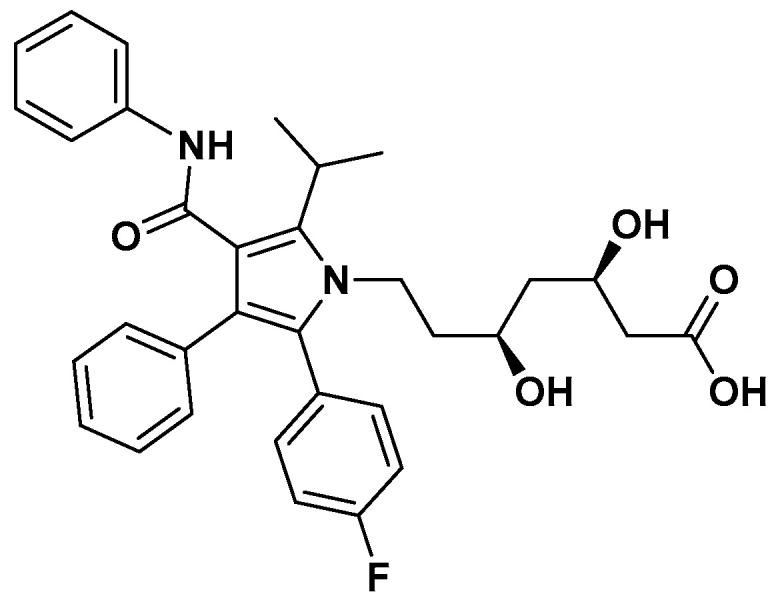
Chemical structure of atorvastatin.

Surineni [195] reported a series of novel carbazole-tethered pyrrole derivatives synthesized using ferric chloride, which showed strong antitubercular activity against *Mycobacterium tuberculosis* H37Rv (MTB) while displaying lower cytotoxicity than other evaluated compounds. Antimicrobial activity has also been observed in bis-pyrrole derivatives derived from hydrazonoyl halides, which demonstrated higher efficacy against Gram-positive bacteria compared to Gram-negative species such as *Pseudomonas aeruginosa* and *Escherichia coli*. One of these compounds was found to be more potent than the standard antifungal Itraconazole against *Aspergillus fumigatus*. Atorvastatin (Figure 6), [196] commercially known as Lipitor, is another example of a pyrrole containing drug used to treat high cholesterol.

Beyond pharmaceuticals, pyrroles are key biosynthetic precursors; the blood pigment heme and the photosynthetic pigment chlorophyll are both derived from the pyrrole-containing intermediate porphobilinogen [197]. Microwave-assisted synthesis enabled the rapid production and evaluation of these compounds, accelerating the discovery of novel anticancer agents. Beyond these activities, pyrroles have shown antiviral, anticoccidial, anti-inflammatory, antipsychotic, anticonvulsant, and antitumor effects. They also function as inhibitors of key biological targets, including histone deacetylases, cyclin-dependent kinases (CDKs), monoamine oxidases, and EGFR tyrosine kinases [198,199].

### 4.2. Anti-Inflammatory Activity

**Scheme 38:**
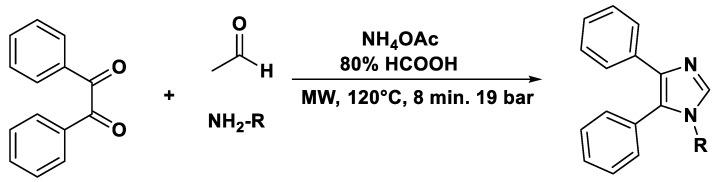
Synthesis of imidazole nucleus.

The pursuit of non-steroidal compounds exhibiting both antifungal and anti-inflammatory properties has been ongoing for many years. Among the wide range of heterocyclic derivatives studied, imidazole stands out as a compound displaying this dual activity. Numerous studies have reported the synthesis of imidazoles and fused imidazole derivatives under MW irradiation conditions. Tripathy et al. developed a one-pot multicomponent reaction to synthesize novel analgesic and anti-inflammatory compounds based on variously substituted imidazole derivatives [200]. All reactants were placed in a long-necked glass vial and subjected to microwave irradiation for 8 min at 120 °C and 19 bar (Scheme 38). In the initial step, ammonium acetate released ammonia under heated, acidic conditions. The generated ammonia then reacted with an aldehyde to form an imine, while another aldehyde molecule interacted with the benzyl carbonyl group to yield a second imine. Cyclization of these two in situ-formed imines resulted in the formation of the imidazole ring, with yields ranging from 60% to 70%. The amines employed in the reaction included aniline, p-isopropylaniline, benzylamine, p-chloroaniline, 1(H)-furfurylamine, and tryptamine.

**Scheme 39:**
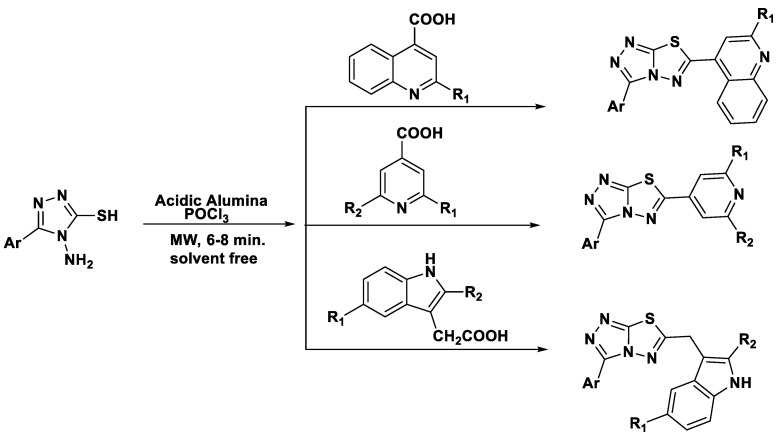
Synthesis of 1,3,4-thiadiazole scaffolds.

MW-assisted methods for the synthesis of functionalized imidazole rings are highly advantageous, particularly for the combinatorial development of new anti-inflammatory and antifungal agents. Beyond antifungal, antiviral, and anticancer activities, imidazole derivatives display a broad range of medicinal properties, including anticonvulsant, antithyroid, antidiabetic, sedative, hypnotic (e.g., Zolpidem), anesthetic, immunosuppressive, anticoagulant, retinoic acid metabolism–blocking, thromboxane synthase inhibitory, and analgesic effects. Mathew et al. reported example of microwave (MW)-assisted, solvent-free synthesis for the preparation of novel biologically active heterocycles [201]. Their research focused on the 1,2,4-triazole and 1,3,4-thiadiazole scaffolds, which share structural similarities with several biologically important compounds, such as thiosemicarbazides and biguanides. This approach enabled the incorporation of two pharmacologically significant nuclei into a single triazolothiadiazole framework. Initially, a solution of 4-amino-3-aryl/alkyl/heteroaryl-substituted-5-mercapto-1,2,4-triazoles the corresponding aromatic acids in POCl_3_ was mixed, adsorbed on alumina, dried, and then subjected to microwave irradiation for a total of 7–8 min (in 30 s intervals), yielding the compounds (Scheme 39).

**Scheme 40:**
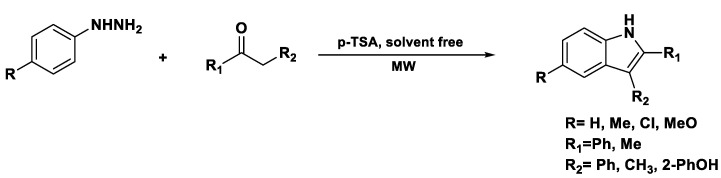
Synthesis of indole scaffold.

The synthesized compounds were then evaluated for their biological activities exhibiting good antimicrobial activity against both Gram-positive and Gram-negative bacteria. Creencia et al. [202] successfully synthesized higher-order substituted indoles via solvent-free, MW-assisted Fischer indole reactions between various substituted phenylhydrazines and ketones in the presence of *p*-toluenesulfonic acid (p-TSA) as a catalyst. MW irradiation eliminated the need for additional solvents, as p-TSA functioned both as a catalyst and as a medium for direct heat transfer to the reactants, resulting in a cleaner reaction and simplified workup (Scheme 40). The reaction was carried out in a single step without isolating the unstable arylhydrazone intermediates, providing higher yields and shorter reaction times compared to conventional non-MW methods, which often produce complex mixtures.

**Figure 7:**
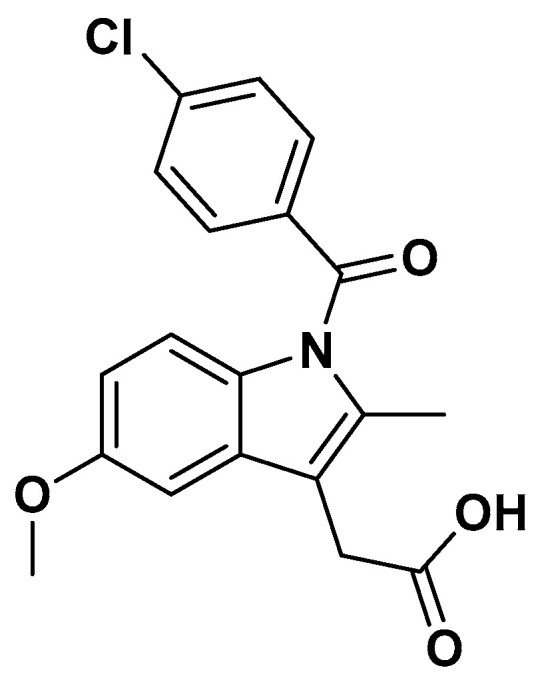
Chemical structure of indomethacin.

The indole heterocycle exhibits a wide range of biological activities, including anticancer, antibacterial, antiviral, anti-inflammatory. It is also employed as an antidepressant, anticholinergic, antiemetic, and antihypertensive agent. Numerous indoles containing compounds are already commercially available, such as the nonsteroidal anti-inflammatory drug Indocid (indomethacin, Figure 7), which acts as a non-selective cyclooxygenase (COX-1 and COX-2) inhibitor for the treatment of chronic conditions like rheumatoid arthritis, ankylosing spondylitis, and osteoarthritis.

Substitution at the second, third, fifth, and sixth positions of the indole ring can further modulate antiviral activity. For example, Enfuvirtide (T-20; Fuzeon), approved by the U.S. FDA in 2003, was the first HIV fusion/entry inhibitor for the treatment of HIV/AIDS. Indole-based drugs also target cancers, such as Alectinib, an ALK inhibitor used in crizotinib-resistant non-small cell lung cancer (NSCLC) adenocarcinoma. More recently, 2-carboxyindole derivatives synthesized by Cury et al. demonstrated selective and promising activity against acute lymphoblastic leukemia cells. Beyond these applications, indoles are widely utilized for antimicrobial, antiemetic, and anti-migraine purposes. Notably, “Triptans,” approved between 1992 and 2001, employ the indole ring as their core scaffold. Indoles can also exhibit potent anticancer effects, as evidenced by drugs such as Sunitinib, used to treat renal cell carcinoma, and Osimertinib, employed in the management of gastrointestinal stromal tumors [203,204,205].

### 4.3. Anticancer Acitivy

**Scheme 41:**
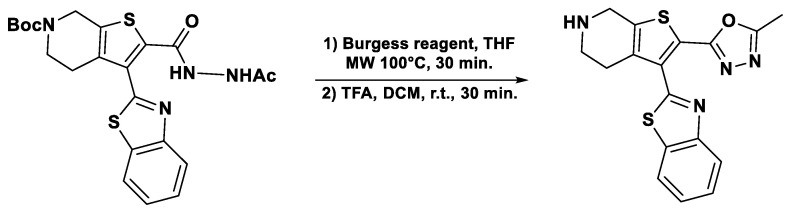
Synthesis of 1,3,4-substituted oxadiazoles.

The global burden of cancer continues to rise, driven primarily by population growth, aging, and the increasing prevalence of cancer-associated lifestyles, particularly in developing countries. The effectiveness of chemotherapy is often constrained by its toxicity toward healthy tissues. Ongoing research seeks to address these limitations, and MW-assisted synthesis offers a greener and more efficient route to generating libraries of *N*-heterocyclic compounds for anticancer drug screening. Rai et al. reported another example of the MW-assisted synthesis of 1,3,4-substituted oxadiazoles, achieved through dehydrative cyclization using the Burgess reagent (Scheme 41) [206].

**Scheme 42:**
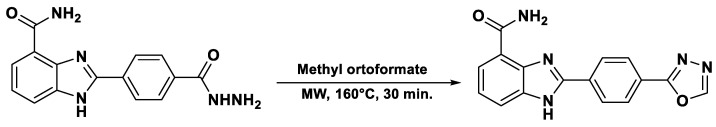
Synthesis of 1,3,4-Oxadiazole derivatives.

We focused on optimizing apurinic/apyrimidinic (AP) endonuclease 1 (APE1) inhibitors incorporating this N-heterocyclic scaffold. APE1 is an appealing target for anticancer therapy, as it is the primary enzyme responsible for removing abasic (AP) sites from DNA in mammals. Since APE1 activity can promote DNA damage, its inhibition may enhance the efficacy of anticancer agents that interact with DNA. MW-assisted syntheses of this compound and various analogues resulted in improved potency of the original lead compound and the identification of new APE1 inhibitors. These small molecules demonstrated inhibitory activity against the purified APE1 enzyme. Furthermore, this class of compounds exhibited favorable in vitro ADME profiles and achieved good plasma and brain exposure levels. Aside from this, 1,3,4-Oxadiazole derivatives were also obtained by Tong et al. from the corresponding benzohydrazide via condensation with methyl orthoformate at 160 °C under MW irradiation for 30 min (Scheme 42).

**Scheme 43:**
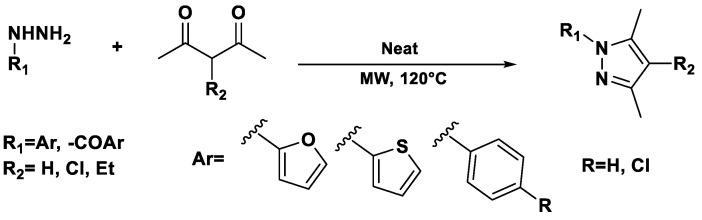
Synthesis of pyrazole scaffolds.

A series of benzoxazole-4-carboxamide compounds were identified as weak inhibitors of poly (ADP-ribose) polymerase (PARP). Subsequently, it was reported the development of a distinct class of PARP inhibitors featuring unsaturated heterocycles attached to a benzimidazole core. PARP-1, a nuclear enzyme belonging to the broader PARP family, becomes activated in response to DNA damage. Upon activation, it cleaves its substrate nicotinamide adenine dinucleotide (NAD^+^) and transfers ADP-ribose units to nuclear target proteins involved in DNA repair [207,208]. This mechanism enables cancer cells to survive and repair DNA lesions caused by chemotherapeutic agents and radiation, thereby evading apoptosis. Consequently, inhibition of PARP-1 has emerged as a promising strategy for anticancer therapy. Several of these newly developed inhibitors exhibited strong activity in both enzymatic and cellular assays, while some also demonstrated favorable pharmacokinetic properties and potent oral in vivo efficacy by enhancing the cytotoxic effects of temozolomide (TMZ) in a mouse xenograft model. The findings indicate that incorporating an unsaturated heterocycle onto the 2-phenyl substituent of the benzimidazole core represents an effective approach for designing potent and orally active PARP-1 inhibitors. In efforts to develop a biologically relevant anticancer pyrazole scaffold, Vaddula [209] introduced a solvent-free, MW-assisted method for the synthesis of tetrasubstituted pyrazoles through the reaction of aryl hydrazines with three-substituted pentane-2,4-diones. MW irradiation enabled the efficient production of various aryl-substituted pyrazoles in high yields without requiring additional purification. This reaction protocol could also be adapted for the synthesis of diazepine derivatives (Scheme 43).

**Scheme 44:**
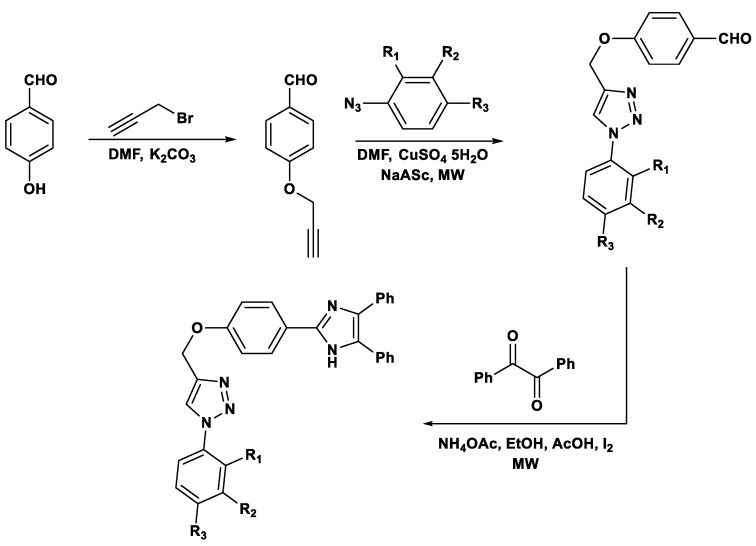
Synthesis of 1,2,3-triazole scaffolds.

The literature highlights that pyrazole derivatives have played a key role in heterocyclic chemistry and serve as important pharmacophores in medicinal chemistry. For instance, Czarnomysy et al. synthesized six novel platinum (II) complexes containing pyrazole, which demonstrated cytotoxic activity against MCF-7 and MDA-MB-231 breast cancer cell lines. The cytotoxic effects were also evaluated against colon carcinoma HCT116 and leukemia K562 human tumor cell lines [210]. Another important anticancer scaffold is the triazole. In recent decades, extensive studies on the structure–activity relationships (SARs) of triazoles have revealed that while substitutions at the first, third, and fifth positions of the triazole ring can all influence biological activity, the most significant effects are generally observed with modifications at the first position. Owing to its ability to accommodate a wide range of substituents, the triazole moiety serves as a highly versatile scaffold in pharmaceutical design [211]. Among triazole derivatives, 1,2,3-triazoles are particularly prominent due to their broad applicability in drug development. These structures can be readily functionalized and combined with a variety of other pharmacologically relevant groups, including those previously discussed, to yield compounds with diverse therapeutic activities. Consequently, MW-assisted synthesis has emerged as an efficient and sustainable strategy for the preparation of novel 1,2,3-triazole derivatives, offering high yields, reduced reaction times, and environmentally benign conditions. N. J. P. et al. synthesized a series of imidazole-linked 1,2,3-triazole derivatives as potential antimicrobial and antioxidant agents. The synthetic route began with the propargylation of 4-hydroxybenzaldehyde to afford the intermediate, which subsequently underwent a copper(I)-catalyzed azide–alkyne cycloaddition (“click” chemistry reaction) with various aryl azides under microwave irradiation to form triazole intermediates. These intermediates were then condensed with benzil and ammonium acetate under microwave conditions, yielding the target compounds (Scheme 44) [212].

**Scheme 45:**
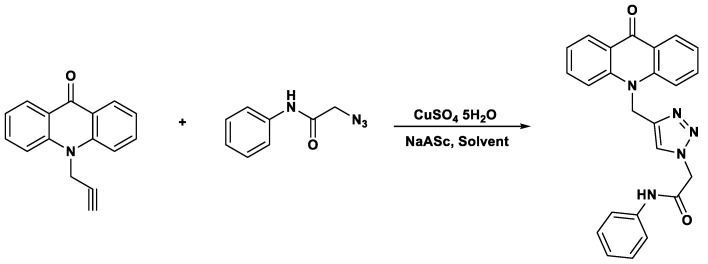
Synthesis of 1,2,3-triazole scaffolds.

Similarly, Amine et al. prepared hybrid molecules containing both acridone and 1,2,3-triazole motifs via copper(I)-catalyzed azide–alkyne cycloaddition under both conventional and microwave conditions. In their approach, 10-(prop-2-yn-1-yl) acridone was reacted with 2-azido-*N*-phenylacetamide in the presence of copper sulfate and sodium ascorbate under varying solvents and heating conditions to produce the desired compound (Scheme 45). This compound served as a representative example to compare the efficiency of conventional and microwave-assisted synthetic methodologies for triazole derivatives. In conventional synthesis, the reaction mixture was stirred at room temperature, whereas in the microwave-assisted method, the reaction proceeded at 200 W and 40–100 °C. Remarkably, microwave-assisted synthesis reduced the reaction time to as little as 1.04% of that required by conventional methods and improved the yield by up to 11% [213].

**Scheme 46:**
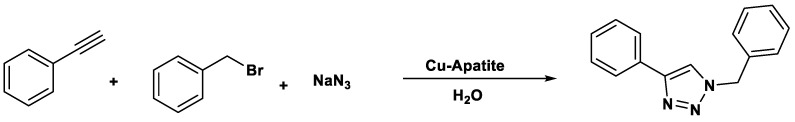
Synthesis of 1,2,3-triazole scaffolds.

Furthermore, Jayaram et al. demonstrated the synthesis of a disubstituted 1,2,3-triazole through the reaction of phenylacetylene, sodium azide, and benzyl bromide using copper apatite as a catalyst in water (Scheme 46). Under conventional heating, the reaction was conducted at 100 °C for 1.5–6 h, whereas the MW-assisted protocol required only 5–20 min at 80 °C and 120 W, highlighting the significant reduction in reaction time and improved efficiency afforded by MW irradiation [214].

**Figure 8:**
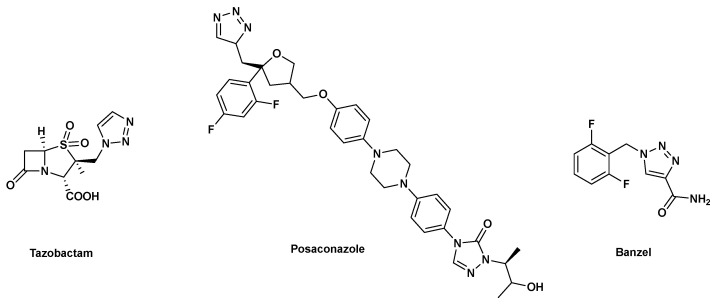
1,2,3-triazole containing pharmaceuticals agents.

Substituted 1,2,3-triazoles are frequently incorporated into bioactive molecules that display a wide spectrum of pharmacological properties, including antiproliferative, anticonvulsant, antimicrobial, antineoplastic, antiviral, analgesic, anti-inflammatory, anticancer, and antimalarial activities [215]. Notably, a melampomagnolide B–triazole derivative has demonstrated potent cytotoxicity against leukemia, melanoma, ovarian, and breast cancer cell lines [216]. Furthermore, 1,2,3-triazole moieties have exhibited significant antibacterial activity against various pathogenic strains, and triazole–pyrimidine–chloroquinoline hybrids have shown strong antiplasmodial effects against *Plasmodium falciparum*. Several 1,2,3-triazole containing drugs (Figure 8) are already in clinical use, exhibiting efficacy across various therapeutic domains, such as *β*-lactam antibiotics (e.g., Tazobactam), antifungal agents (e.g., Posaconazole), and antiepileptic drugs (e.g., Banzel) [217].

### 4.4. Antibiotic Activity

Lactams are a class of antibiotics widely utilized across various fields, including drug discovery and polymer manufacturing [218]. The synthesis of lactams has long been a central topic in organic chemistry and remains an area of active research. Traditionally, industrial production of most lactams involves multistep reactions that require harsh reagents and elevated temperatures [219]. However, MW-assisted protocols may offer advantageous alternatives, allowing these reactions to proceed under milder conditions while achieving higher yields. The underlying chemistry involves the nucleophilic attack of a reactive species such as the hydroxyl group of a serine residue on the strained lactam ring. In particular, the bicyclic *β*-lactam ring present in amoxicillin is highly susceptible to hydrolytic cleavage of the amide bond, yielding a secondary amine and an ester. This disruption interferes with normal cellular processes, ultimately leading to cell death.

**Scheme 47:**
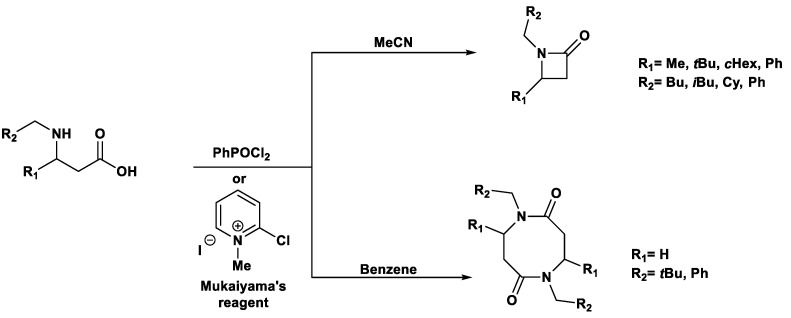
Synthesis of *β*-amino lactam derivates.

Hernández-Vázquez et al. [220] carried out the reaction of several *β*-amino acids by activating the carbonyl group with the organophosphorus reagent phenylphosphonic dichloride (PhPOCl_2_) or Mukaiyama’s reagent under microwave (MW) irradiation to synthesize *β*-lactams or cyclic *β*-dipeptides (Scheme 47). When PhPOCl_2_ was used in benzene, the reaction proceeded via two possible pathways depending on the β-substituent. In contrast, using Mukaiyama’s reagent, *N*-substituted *β*-amino acids yielded either *β*-lactams, cyclic *β*-dipeptides, or a mixture of both, depending solely on the solvent. In benzene, the formation of cyclic *β*-dipeptides was favored, whereas reactions conducted in acetonitrile predominantly produced *β*-lactams.

The observed solvent-dependent selectivity under MW irradiation when using Mukaiyama’s reagent may be attributed to a thermal effect. Although both benzene and acetonitrile are relatively nonpolar and do not efficiently absorb microwave energy, the heating process in MW-assisted reactions is governed by the polarity of the reagents involved. Mukaiyama’s reagent, being a pyridinium salt, can interact with microwaves through ionic conduction, leading to selective heating of the reaction medium. This phenomenon often described as a “molecular radiator” effect creates microscopic hot spots within the reaction vessel, thereby enhancing the selectivity of the chemical transformation. The antibiotic activity of *β*-lactams is well established. These compounds act by inhibiting D-alanyl-D-alanine carboxypeptidases, thereby blocking the biosynthesis of the peptidoglycan layer in bacterial cell walls and ultimately halting bacterial cell division. Owing to their structural resemblance to the D-alanyl-D-alanine terminus, *β*-lactam antibiotics can irreversibly acylate the active site of the transpeptidase enzyme, disrupting peptidoglycan cross-linking and promoting bacterial cell wall hydrolysis.

Although a wide variety of *β*-lactam antibiotics are currently available and highly effective, the growing problem of antibiotic resistance among pathogenic bacteria underscores the urgent need for novel *β*-lactam derivatives. Beyond their well-known antibacterial activity, *β*-lactams exhibit remarkable structural versatility. Modifications to their pharmacophoric groups have led to compounds with diverse biological activities, including thrombin inhibition, antihyperglycemic, anticancer, antiproliferative, anti-HIV, antifungal, antitubercular, and antioxidant properties. Further structural elaboration of *β* -lactams into spirocyclic scaffolds has yielded molecules with additional biological activities such as antidiabetic, anti-inflammatory, analgesic, anticancer, and peptidomimetic effects. These derivatives have also been reported to inhibit acetyl-CoA cholesterol acyltransferase and picornaviruses. More recently, the combination of D-serine with *β*-lactam antibiotics has demonstrated promising antibacterial activity against *Staphylococcus aureus* [221,222,223].

### 4.5. Drugs for Cardiovascular Activity

**Scheme 48:**
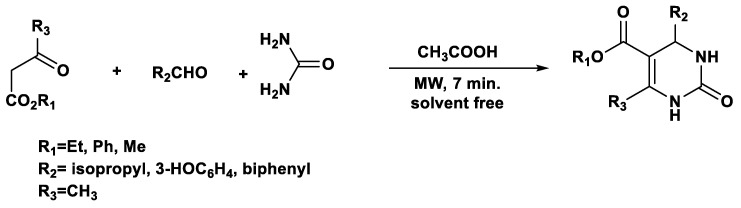
Synthesis of 3,4-dihydropyrimidonenes.

Cardiovascular diseases remain the primary cause of mortality in many developing countries and a major contributor to disability in industrialized regions. Moreover, severe cardiac disorders often exhibit mortality rates comparable to those of the most aggressive malignancies. Numerous *N*-heterocyclic scaffolds relevant to cardiovascular drug discovery have been efficiently synthesized through MW-assisted methods. Sujatha et al. [224] reported the MW-assisted synthesis of 3,4-dihydropyrimidinones (DHPMs), as illustrated in Scheme 48. These compounds were obtained by heating 1,3-dicarbonyl compounds, urea, and aromatic aldehydes in acetic acid under MW irradiation, using a modified domestic oven. The reactions, monitored by TLC, were completed within 5–7 min (pulse rate 40 s, power 30%). DHPMs, also known as Biginelli products, exhibit a wide range of biological activities. Their structural resemblance to Hantzsch-type dihydropyridines, potent Ca^2+^ channel blockers, renders them particularly attractive in cardiovascular medicinal chemistry. The cardiotonic activity of the synthesized DHPMs was evaluated on an isolated perfused frog heart model and compared with that of digoxin under identical conditions.

**Scheme 49:**
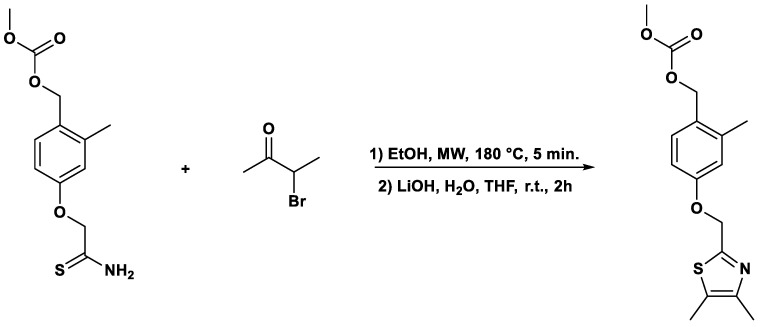
Synthesis of thiazole derivatives.

In another study, Epple et al. [225] achieved the rapid formation of a thiazole ring through MW-assisted Hantzsch condensation between a thioamide intermediate and α-bromoketone at 180 °C for 5 min (Scheme 49). The resulting thiazole derivatives were shown to fit within a large hydrophobic pocket of peroxisome proliferator-activated receptors (PPARs), which regulate genes involved in energy homeostasis. These findings identified the compounds as potential leads for the treatment of metabolic syndrome, a condition that markedly increases cardiovascular risk. Over 150 analogues were synthesized using this MW-assisted cyclization protocol, leading to a novel series of selective PPARδ agonists. Several derivatives are currently under further investigation to elucidate the role of PPARδ in glucose and lipid metabolism and to evaluate their therapeutic potential in metabolic syndrome-related disorders.

### 4.6. Drugs for Central Nervous Disease

**Scheme 50:**
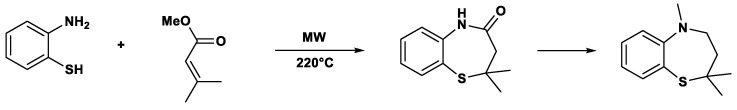
Synthesis of benzodiazepine scaffold.

Among the most widely used therapeutic classes, drugs acting on the central nervous system (CNS) account for approximately 15% of the total. The exceptional capacity of heterocyclic frameworks to function both as biomimetic structures and as active pharmacophores has significantly contributed to their distinctive role as essential components of many pharmaceutical agents. MW irradiation is increasingly emerging as a crucial tool for optimizing key synthetic steps in the development of CNS-active drugs. Wu et al. [226] focused on identifying new antagonists for the neuropeptide Y5 (NPY5) receptor, which plays a key role in several biological processes within both the central and peripheral nervous systems. NPY5 antagonists, for instance, have potential therapeutic applications in regulating food intake and managing obesity. Although moderately potent NPY5 antagonists containing a benzothiazepinone–glycinamide scaffold was previously known, these compounds exhibited limited stability and poor pharmacokinetic properties. To address these issues, it was reported an example of microwave-assisted cyclization. Specifically, the condensation of 2-aminothiophenol with methacrylate derivative yielded benzothiazepinone, which were subsequently reduced to obtain the corresponding benzothiazepine (Scheme 50). Further functionalization of this core structure produced a series of compounds that demonstrated promising NPY5 antagonistic activity. In addition, the authors conducted an extensive structure–activity relationship (SAR) study to better understand the molecular features influencing bioactivity.

**Scheme 51:**
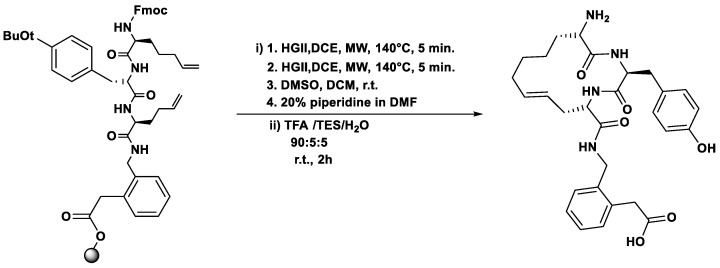
Synthesis of dioxo-1,4-diazacyclotetradec-7-ene derivative.

The benzodiazepine nucleus, on the other hand, is a well-established pharmacophore known for its broad spectrum of therapeutic and pharmacological properties. Numerous benzodiazepine derivatives are widely used as anxiolytic, antidepressant, sedative, hypnotic, tranquilizing, anticonvulsant, antihistaminic, analgesic, and anti-inflammatory agents. Another example of MW-assisted reaction is reported by Andersson et al. [227] that utilized an olefin ring-closing metathesis (RCM) reaction to carry out a macrocyclization, leading to the formation of a substituted dioxo-1,4-diazacyclotetradec-7-ene derivative (Scheme 51). The compound was synthesized manually using solid-phase peptide synthesis (SPPS) with the 9-fluorenylmethoxycarbonyl (Fmoc) protection strategy, followed by side chain to side chain cyclization through ring-closing metathesis (RCM). In fact, the cyclization was carried out on a preparative scale using the Hoveyda–Grubbs second generation catalyst (HGII) at 150 °C for 5 min, followed by a second addition of the catalyst and repetition of the reaction.

This procedure yielded a potent inhibitor of insulin-regulated aminopeptidase (IRAP), an enzyme localized in brain regions associated with memory and learning. The study reported the design, synthesis, and biochemical evaluation of novel 13- and 14-membered macrocyclic tripeptide analogues of angiotensin IV. It was shown that replacing the disulfide bridge in the *N*-terminal macrocyclic segment with a carbon–carbon linkage was well tolerated. This molecule is potent, selective (*K*_i_ = 4.1 nM), and it represents a promising lead compound for further optimization.

## 5. MW-Assisted Green Chemistry in Peptide Synthesis

Peptides play key roles in medicinal chemistry being used in therapeutics and diagnostic agents. Around the early 1990s, Green Chemistry, also referred to as sustainable chemistry, was introduced to drastically minimize pollution [228,229].

**Scheme 52:**
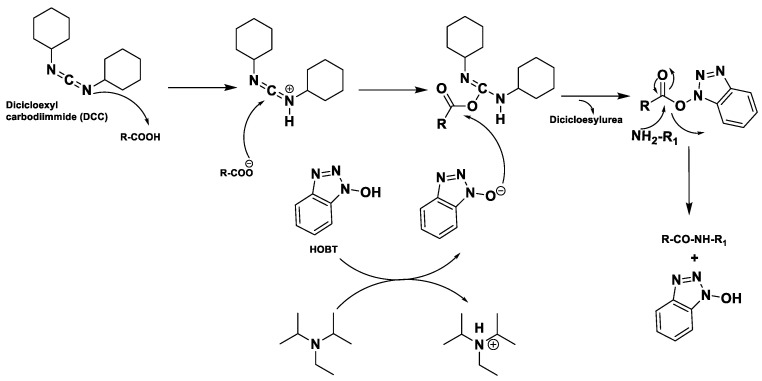
Coupling reaction with I generation coupling reagents.

Coupling reagents are essential in peptide synthesis to ensure that the coupling reaction proceeds to completion. Initially, carbodiimide-based reagents such as DCC (*N*,*N*′-dicyclohexylcarbodiimide) (Scheme 52) were among the first to be used and remain in use today, even if with the introduction of more advanced alternatives. Nevertheless, carbodiimides are associated with health hazards, as they can cause skin irritation and are considered potentially carcinogenic.

**Scheme 53:**
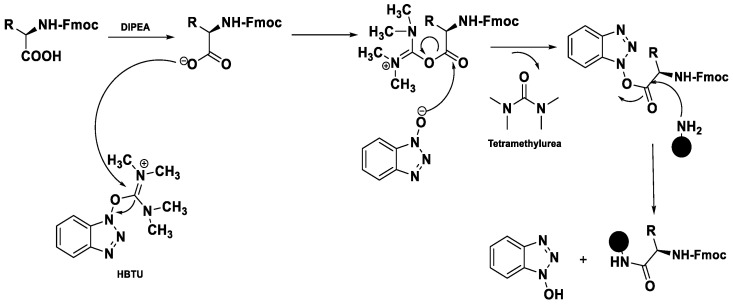
Coupling reaction with II generation reagents.

In most recent Fmoc-based solid-phase peptide syntheses (SPPS), next-generation benzotriazole-derived coupling reagents such as HBTU and HATU are commonly used in DMF (Scheme 53). To minimize side reactions like guanidination of the *N*-terminal amine, which is particularly associated with aminium/uronium-type reagents, the coupling reagent is typically used in slightly lower concentrations than the amino acid. Two major challenges during the coupling step are incomplete coupling and racemization. To mitigate racemization, additives like HOAt and HOBt are frequently added to the reaction mixture. Additional strategies such as prolonged reaction time, using stronger coupling agents, or applying microwave irradiation may also improve outcomes [230].

**Scheme 54:**
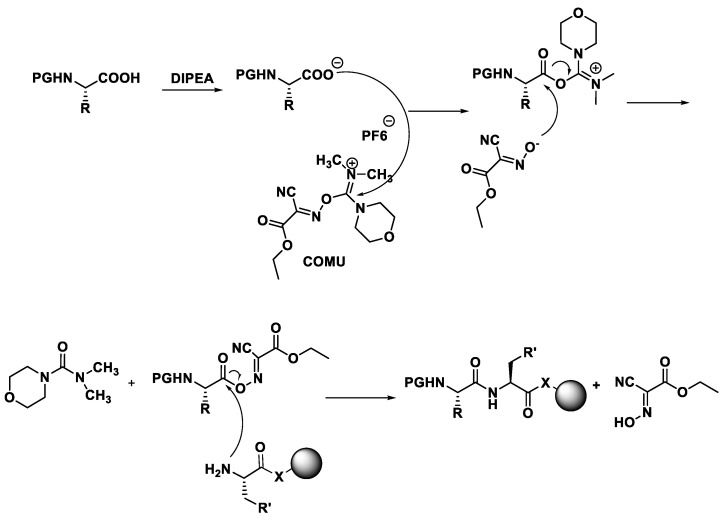
Coupling reaction with III generation reagents.

The hazardous nature of HOBt and HOAt, combined with the toxicity of HATU, prompted many research teams to explore and publish their findings on third-generation coupling reagents and racemization inhibitors [230]. Many researchers have successfully used COMU [(1-cyano-2-ethoxy-2-oxoethylidenaminooxy) dimethylamino-morpholino-carbenium hexafluorophosphate] as a coupling reagent in place of HBTU and HATU, often achieving excellent results. In some instances, coupling with COMU led to higher yields compared to HATU (Scheme 54). Although initial concerns regarding COMU’s stability in DMF and other organic solvents due to its potential hydrolysis, recent studies have addressed these issues, demonstrating COMU’s stability in more environmentally friendly solvents. Differential scanning calorimetry (DSC), a method used to analyze decomposition kinetics, confirmed that COMU’s morpholonium and oxime-based structure is non-autocatalytic; these findings further affirmed the safety of COMU, significantly reducing the risk of a thermal runway and explosion. COMU has also been successfully applied in amide bond formation via standard batch synthesis and also in MW-assisted peptide synthesizers, MW-irradiation being perfectly compatible with oxyma-based uronium-type coupling reagent like COMU [231].

### 5.1. Green Solvents in Peptide Synthesis

**Figure 9:**
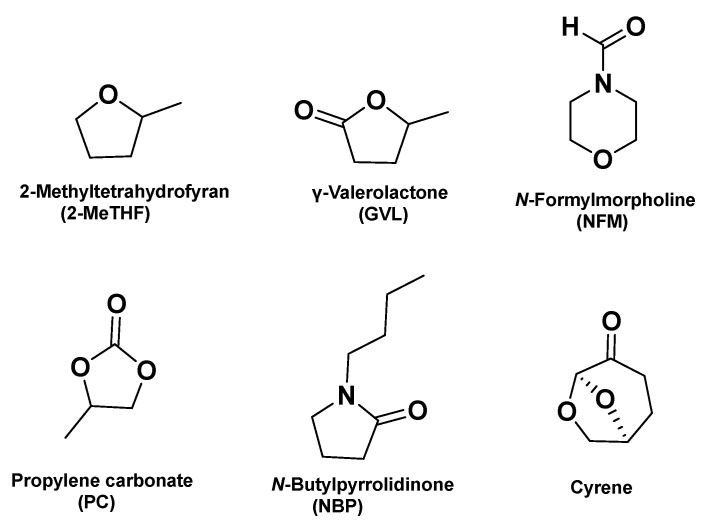
Green solvents for peptide synthesis.

Solvents play a crucial role in chemical reactions and are one of the primary components to consider from a green chemistry standpoint [232]. The importance of solvents is emphasized in the principles of Green Chemistry, which is focused on designing products and processes that reduce or eliminate hazardous substances [233]. The scientific community has increasingly focused on this area, leading to the development of several solvent selection guides by pharmaceutical companies and collaborative initiatives. These guides assess the Environmental, Health, and Safety (EHS) characteristics of solvents and categorize them as recommended, problematic, hazardous, or highly hazardous. Problematic solvents may still be used in lab-scale or pilot-scale research but require specific waste handling measures [234,235]. Hazardous solvents are tightly regulated during scale-up processes, while highly hazardous ones should be avoided even at the laboratory level. Therefore, finding replacements for these hazardous categories is essential. Green solvents are essentially safer alternatives with better EHS profiles or those derived from renewable sources [236]. Currently, DCM, DMF, and NMP are the most used solvents in Solid Phase Peptide Synthesis (SPPS). Dimethylformamide (DMF) is commonly utilized in the pharmaceutical industry due to its low volatility, small molecular size, and chemical characteristics such as its role as an electron donor and its ability to promote complex formation. However, concerns over its toxicity began to surface prominently in the 1980s, following numerous reported cases of harmful exposure. Research has shown that DMF can enter the human body through skin contact or inhalation. Notably, DMF has been linked to hepatotoxic effects, which may result in liver damage [237,238]. However, developing more environmentally friendly solvents suitable for SPPS remains a priority. In 2013, Watson and colleagues explored various green solvents to replace DMF and DCM in solution-phase peptide synthesis. Their study examined coupling reactions involving alkyl and aryl acids with amines and found that dimethyl carbonate, 2-methyltetrahydrofuran (2-MeTHF), and ethyl acetate (EtOAc) performed well, especially when using COMU as a coupling agent. Further research by Lopez and colleagues at Novartis evaluated N-butylpyrrolidinone (NBP) as a green alternative to DMF (Figure 9). NBP has favorable properties: it is biodegradable, non-mutagenic, and not toxic to reproduction. In peptide synthesis, it showed effective swelling of polystyrene resins and could dissolve Fmoc amino acids comparably to DMF. Lastly, cyrene has emerged as a bio-based solvent for amide bond formation. Made from renewable resources, it has shown promising results in synthesizing dipeptides using HATU as a coupling reagent, with high yields [239,240].

In addition, the use of cyrene has also been found in microwave-assisted synthesis, in particular as a substitute for DMF, ensuring better performance by being able to heat to higher temperatures [241]. Several studies attested in addition the good MW absorptivity also of propylene carbonate (PC) due to its dielectric constant of 64 [242].

### 5.2. Deep Eutectic Solvents (DESs)

**Figure 10:**
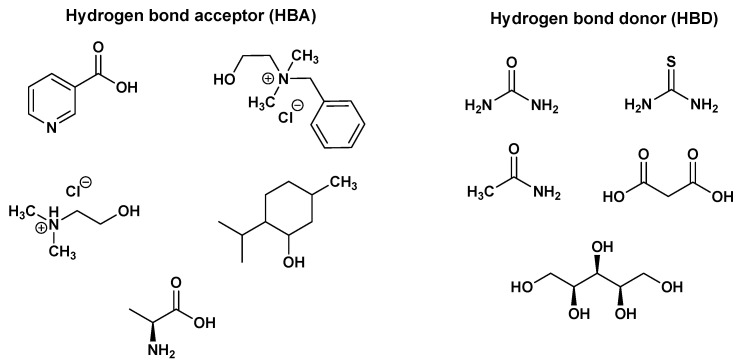
Deep Eutectic Solvents.

Deep eutectic solvents (DESs) are a novel and promising category of environmentally friendly solvents, notable for their substantially lower melting points relative to those of their individual constituents. While they share several features with ionic liquids, DESs tend to offer greater sustainability and are more economical to synthesize (Figure 10) [243].

DESs are characterized by several features including high polarity which plays a key role in their chemical behavior. The interaction between the hydrogen bond acceptor (HBA) and donor (HBD) allow these solvents to interact effectively with polar and/or charged species, promoting the formation of new chemical bonds. They possess high viscosity which leads to prolonged interaction times and can improve overall the conversion yields [244,245]. Additionally, their viscosity contributes to enhanced chemical and thermal stability, limiting both volatility and degradation. DESs are characterized by very low vapor pressure, making them significantly less volatile than traditional organic solvents. They are generally considered to be less harmful than many conventional solvents, aligning well with the principles of green chemistry and sustainable processing. Often synthesized from natural or renewable substances, DESs are typically biodegradable, making them more compatible with environmental protection goals. Finally, DESs are highly effective in dissolving and stabilizing a broad panel of compounds, including transition metal catalysts. This capability is largely due to their strong hydrogen-bonding network and to their high polarity, which makes them solvents suitable for polarizing also in the presence of microwave irradiation, rapidly converting the electromagnetic energy into heat [246,247,248,249,250].

## 6. Conclusions and Perspectives

Over the years, microwave-assisted synthesis of heterocycles had a significant environmentally sustainable impact in various fields, including medicinal chemistry, polymer synthesis, and material science. Many studies explored heterocycle synthesis using green solvents and catalyst-free protocols, such as the microwave-assisted Fischer indole synthesis, which was scaled from batch to continuous mode. The beneficial effects of microwaves on heterocycle synthesis are particularly relevant in pharmaceutical chemistry, where reduced reaction times and high efficiency are critical. Numerous microwave-assisted multicomponent reactions have been reported, including the Passerini reaction and three-component spiro-oxindole synthesis. These methods demonstrated that microwave irradiation, particularly when combined with continuous-flow systems, enhances reaction efficiency, and selectivity. Ultimately, MAOS has revolutionized the field, offering rapid, high-yielding, and environmentally friendly alternatives to conventional synthetic methods. Future research will likely continue expanding the applications of microwave chemistry in various scientific and industrial domains.

## Figures and Tables

**Table 1:** Dielectric constant of solvents.

Solvent	Dielectric Constant (ε′)	Dipole Moment	Dissipation Factor (tan δ)	Boiling Point (°C)	Temperature in a Closed Container
Acetone Acetonitrile Ethanol	20.7	-	-	56	164
	37.5	-	-	82	194
	24.3	1.96	2.500	78	164
Hexane Methanol Water	1.89			69	-
	32.6 78.3	2.87 2.30	6.400 1.570	65 100	151 -

**Table 2:** Tangent δ scale.

Solvent	Tan δ	Solvent	Tan δ	Solvent	Tan δ
Ethylene glycol Ethanol DMSO	1.350	2-Butanol	0.447	Chloroform	0.091
	0.941	Dichlorobenzene	0.280	Acetonitrile	0.062
	0.825	NMP	0.275	Ethyl acetate	0.059
2-Propanol Formic acid	0.799	Acetic acid	0.174	Acetone	0.054
	0.722	DMF	0.161	THF	0.047
Methanol Nitrobenzene 1-Butanol	0.659	Dichloroethane	0.127	Dichloromethane	0.042
	0.589	Water	0.123	Toluene	0.040
	1.350	Chlorobenzene	0.101	Hexane	0.020

**Table 3:** Hydrogenation of various substrates.

Reactant	Product	Time (minutes)	Yield (%)
** 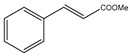 **	** 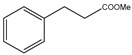 **	**3**	**99**
** 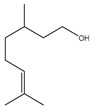 **	** 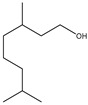 **	**5**	**99**
** 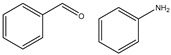 **	** 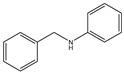 **	**20**	**89**
** 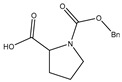 **	** 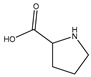 **	**5**	**89**
** 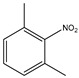 **	** 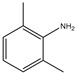 **	**15**	**99**

**Table 4:** Microwave-assisted CM and RCM reactions.

Olefin	CM-Partner	Product	Conditions	Catalyst	Yield%
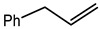	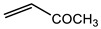	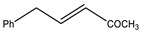	2.5 mol% of catalyst CH_2_Cl_2_, 12h	II gen. Grubbs Hoveyda-Grubbs Zhan	**99** **99**
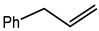	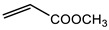	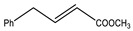	2.5 mol% of catalyst CH_2_Cl_2_, 5h	II gen. Grubbs Hoveyda-Grubbs Zhan	**99** **99** **99**
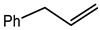	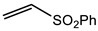	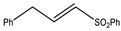	2.5 mol% of catalyst CH_2_Cl_2_, 12h	II gen. Grubbs Hoveyda-Grubbs Zhan	**43** **71** **62**
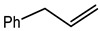	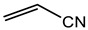	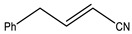	2.5 mol% of catalyst CH_2_Cl_2_, 5h	II gen. Grubbs Hoveyda-Grubbs Zhan	**51** **67** **59**
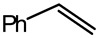	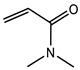	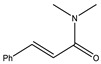	5 mol% of catalyst CH_2_Cl_2_, MW, 100 °C, 30 min	II gen. Grubbs Hoveyda-Grubbs Zhan	**30** **65** **45**

## Data Availability

All data supporting the findings in this review paper are publicly available and can be accessed through the cited literature. The authors confirm that the data supporting the findings of this study are available within the article.

## Abbreviations

The following abbreviations are used in this manuscript:

MAOS	Microwave-assisted organic synthesis
MW	Microwaves
LET	Light emitting technology
PTFE	Politetrafluoroethylene
DBSA	p-Dodecylbenzenesulfonic acid
INH	Isoniazid
PTSA	p-toluenesulfonic acid
NMP	*N*-methyl-2-pyrrolidone
RCM	*N*-methyl-2-pyrrolidone
RCM	Ring-closing metathesis
CM	Closing metathesis
HGII	Hoveyda-Grubbs II generation catalyst
HMF	5-hydroxymethylfurfural
PEG	Polyethylene glycol
VOCs	Volatile organic compounds
PAT	Process analytical technology
DMSO	Dimethyl sulfoxide
SEM	Scanning Electron Microscopy
EDX	X-ray Spectroscopy
PID	Proportional integral derivative
ML	Machine learning
PID	Proportional-integral-derivative
MCRs	Multicomponent reaction
